# Does Topology Dictate the Incidence of the Twist‐Bend Phase? Insights Gained from Novel Unsymmetrical Bimesogens

**DOI:** 10.1002/chem.201604030

**Published:** 2016-11-15

**Authors:** Richard J. Mandle, John W. Goodby

**Affiliations:** ^1^ Department of ChemistryUniversity of YorkYorkYO10 5DDUK

**Keywords:** chirality, liquid crystals, molecular shape, soft matter, symmetry

## Abstract

We prepared a significant number of unsymmetrical liquid‐crystalline dimers that exhibit the twist‐bend nematic phase; a state of matter that exhibits spontaneous breaking of mirror symmetry and, for some materials, a microsecond electrooptic response. A number of novel unsymmetrical bimesogens were synthesized and in comparing their thermal behaviour to previous literature examples, we have uncovered an unexpected relationship between the thermal stability of the nematic and N_TB_ phases. This relationship demonstrates that molecular shape dictates the incidence of this fascinating phase of matter and leads us to speculate as to the existence of “twist‐bend nematic phases” on length scales beyond those of the molecule.

## Introduction

The observation of a lower‐temperature mesophase lacking lamellar or positional molecular order (i.e., a nematic‐like mesophase),^1^ later identified as the “twist‐bend nematic” phase (N_TB_),^2^, ^3^, ^4^, ^5^, ^6^, ^7^, ^8^, ^9^, ^10^ has motivated many studies of the properties of this intriguing phase of matter through a wide range of techniques.^11^, ^12^, ^13^, ^14^, ^15^, ^16^, ^17^, ^18^ Although the helicoidal model of the N_TB_ phase, proposed independently by Meyer and Dosov,^19^, ^20^ is supported by experimental data, alternate models have also been suggested.^21^, ^22^, ^23^, ^24^ With the exception of Vanakaras et al.,^23^ theoretical treatments have not accounted for direct isotropic to twist‐bend nematic phase transitions, a scenario first reported in a mixed dimer/chiral‐dopant system.^25^ Initially, it was believed that this mesophase was only found in methylene‐linked dimers. However, observations of the N_TB_ phase in bent‐core systems,^8^ ether‐linked dimers,^26^, ^27^ trimers,^28^, ^29^, ^30^, ^31^ oligomers^32^ and even polymers^33^ hint at this mesophase being universal rather than what was perhaps first thought. A comprehensive structure property relationship remains elusive despite the ever growing number of compounds reported in the literature to exhibit this mesophase.^35^, ^36^, ^37^, ^38^, ^39^, ^40^, ^41^, ^42^, ^43^, ^44^

It is well known that polar functional groups can, through dipole‐dipole or quadrupolar interactions, lead to antiparallel pairing of molecules; the extent to which this occurs is functional‐group dependent.^45^, ^46^, ^47^ Previously, we explored how this manifests in a small number of unsymmetrical bimesogens with two “polar” terminal groups.^41^ In this report, we expand on this pervious work, introducing a large number of new unsymmetrical polar bimesogens with differing polar functional groups, as well as preparing several bimesogens possessing one polar and one non‐polar terminal group. In all cases, the mesogenic units employed in this study are appropriately substituted phenyl benzoates with a nonamethylene spacer and methylene linking groups. Ultimately, in our comparative analysis of these novel materials with previous symmetrical polar or apolar materials, we discovered an unexpected relationship between the thermal stability of the nematic and N_TB_ phase, which we will now describe.

## Results and Discussion

**Scheme 1:**
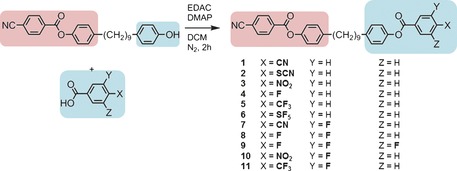


**Scheme 2:**
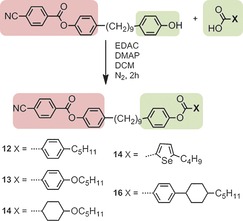


The synthesis and characterisation of compounds **1**–**6** has been reported previously.^41^ Compounds **7**–**11** were synthesised, as shown in Scheme 1, by the EDAC/DMAP‐mediated (EDAC: 1‐ethyl‐3‐(3‐dimethylaminopropyl)carbodiimide, DMAP: 4‐dimethylaminopyridine) Steglich esterification between [4‐{9‐(4‐hydroxyphenyl)nonyl}phenyl 4‐cyanobenzoate], prepared as described previously,^29^ and an appropriate benzoic acid. Compounds **12**–**16**, which contain one polar and one apolar rigid unit, were prepared by the EDAC/DMAP‐mediated Steglich esterification between [4‐{9‐(4‐hydroxyphenyl)nonyl}phenyl 4‐cyanobenzoate] and a series of non‐polar benzoic and carboxylic acids, as shown in Scheme 2.

**Figure 1:**
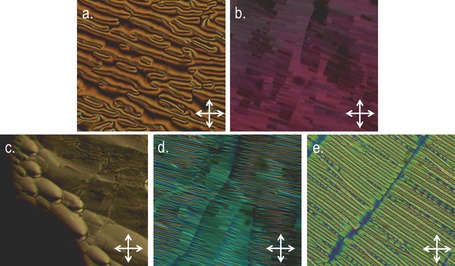
Photomicrographs (×100) on untreated glass slides of: a) the schlieren texture of the nematic phase of **3** at 107.5 °C, b) the blocky texture of the N_TB_ phase of compound **7** at 93.5 °C, c) parabolic defects in an uncovered droplet of the N_TB_ of compound **10** at 77.0 °C, d) the stripe and blocky textures of the N_TB_ phase along with visible domain boundaries for compound **8** at 61.1 °C, and e) the rope texture of the N_TB_ phase of compound **7** at 91.0 °C confined in a 5±0.1 μm cell with antiparallel polyimide alignment layers.

**Figure 2:**
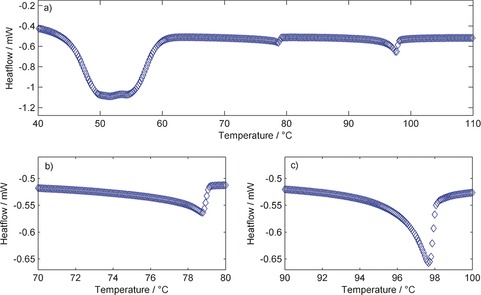
DSC thermogram of compound **10** obtained at 10 °C min^−1^ on the first cooling cycle (a), with expansions between 70–80 °C showing the nematic to N_TB_ transition (b), and between 90–100 °C showing the nematic to isotropic transition (c). The large “bump” with an onset of around 60 °C in (a) corresponds to the sample recrystallizing rather than a transition to a mesophase.

The transition temperatures of compounds **1**–**11**, which bear two polar “core” units, were determined by a combination of polarised optical microscopy (POM) and differential scanning calorimetry (DSC). As shown in Table 1, all eleven materials were found to exhibit nematic and twist‐bend nematic mesophases. The identification of the lower temperature mesophase as the twist‐bend nematic is straightforward due to its characteristic optical textures (Figure 1) and the shape of the peak obtained in DSC thermograms (Figure 2).^9^

**Table 1:** Transition temperatures [°C] for compounds **1**–**11**.^39^, ^41^ Transitions in parentheses are monotropic, that is, they occur below the melting point of the material in question.

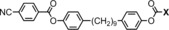								
Compound	X	Cr		N_TB_		N		Iso
**1**	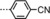	•	157.6	(•	114.5	•	146.6)	•
**2**	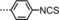	•	95.6	•	100.0	•	123.8	•
**3**	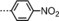	•	115.4	(•	100.5)	•	124.9	•
**4**	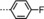	•	86.2	(•	78.2)	•	95.9	•
**5**	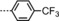	•	110.0	(•	69.6	•	78.3)	•
**6**	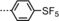	•	102.2	(•	61.1	•	72.8)	•
**7**	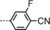	•	112.6	(•	95.0)	•	120.7	•
**8**	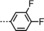	•	83.2	(•	63.8	•	79.0)	•
**9**	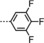	•	93.8	(•	46.0	•	60.0)	•
**10**	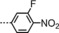	•	92.6	(•	78.8)	•	97.6	•
**11**	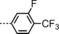	•	105.7	(•	51.4	•	57.5)	•

With the exception of compound **2**, the N_TB_ phases exhibited by **1**–**11** were monotropic, although the nematic phases of several materials (**2**, **3**, **4** and **10**) were enantiotropic. Compared to the symmetric parent material of the series, compound **1**, the unsymmetrical materials all exhibit lower melting points, clearing points and nematic to N_TB_ transition temperatures. Representative photomicrographs of the nematic and N_TB_ mesophases of compounds **3**, **7**, **8** and **10** are given in Figure 1. A DSC thermogram of compound **10** is given in Figure 2, the nematic to isotropic transition was first order with the N_TB_‐N transition being weakly first order, as reported previously.^9^

The nematic mesophases of all compounds were identified based on their schlieren textures, whereas the twist‐bend phase exhibited by **1**–**11** was identified based on the characteristic textures (block, rope, parabolic, stripe; see Figure 1 b–e for representative examples) exhibited by this phase. The associated enthalpies and entropies of transition for compounds **1**–**11** were determined by DSC at a heat/cool rate of 10 °C min^−1^ and are presented in Table 2. Values are given as the mean of nine cycles along with corresponding standard deviations.

**Table 2:** Associated enthalpies of transition [kJ mol^−1^] with corresponding standard deviations (SD), dimensionless entropies of transition and scaled N/N_TB_ transition temperatures for compounds **1**–**11**.^38^, ^41^

Compound	Δ*H* [kJ mol^−1^]				Δ*S*/*R*		*T* ${}_{N{}_{\mathrm{TB}}\text{-}N}$ */T*_N‐Iso_
	N_TB_‐N	SD	N‐Iso	SD	N_TB_‐N	N‐Iso	
**1**	0.402	0.005	1.728	0.008	0.012	0.495	0.924
**2**	0.244	0.003	0.697	0.005	0.131	0.192	0.940
**3**	0.092	0.042	0.289	0.003	0.046	0.087	0.939
**4**	0.074	0.009	1.015	0.005	0.045	0.331	0.952
**5**	0.473	0.022	0.315	0.003	0.271	0.108	0.975
**6**	0.480	0.090	0.970	0.102	0.286	0.337	0.966
**7**	0.142	0.006	1.312	0.004	0.134	0.401	0.935
**8**	0.254	0.031	0.245	0.002	0.036	0.498	0.957
**9**	0.724	0.007	0.648	0.003	0.189	0.224	0.958
**10**	0.235	0.003	0.741	0.002	0.116	0.200	0.951
**11**	0.450	0.014	0.810	0.006	0.105	0.186	0.982

**Figure 3:**
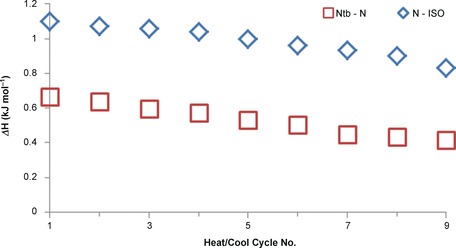
Associated enthalpies of transition (Δ*H* [kJ mol^−1^]) for the nematic to isotropic (**□**) and N_TB_ to nematic (**◊**) phase transitions of compound **6** as a function of DSC cycle number.

With the exception of compound **2** (X=ArSCN) the associated enthalpies of both the N‐Iso and N_TB_‐N transition are highly reproducible, with only small deviations from the mean. Ultimately, and as reported previously,^38^, ^39^, ^40^ we find no correlation between the dipole moment of the mesogenic unit and the onset temperature, associated entropy or associated enthalpy of either the N‐Iso or N_TB_‐N transitions. In the case of compound **6**, the associated enthalpies of both the N‐Iso and N_TB_‐N transitions were observed to decline with each successive heat/cool cycle, as shown in Figure 3. Thus, the large standard deviation values can likely be attributed to thermal decomposition of the sample. No decomposition was apparent by either POM or DSC studies of the other compounds in Table 1 and so the decomposition is believed to be due to the instability of the isothiocyanate group.

**Figure 4:**
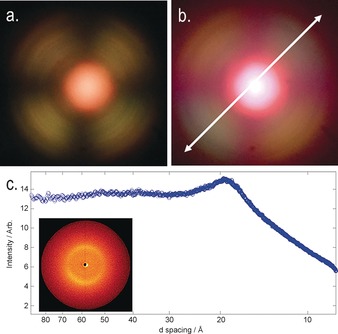
a) Conoscopic figure in the N_TB_ phase of **7** confined in a 5±0.1 μm cell with antiparallel buffed polyimide alignment layers (planar anchoring) at approximately 80 °C without a waveplate; b) the same image as in (a) but with a λ‐plate inserted into the optical path, the white arrow denoting the slow axis of the λ‐plate; c) plot of scattered X‐ray intensity as a function of *d*‐spacing (in the range of 8–95 Å, utilised with a sample‐to‐detector distance of 300 mm) for a partially aligned sample of compound **10** in the N_TB_ phase at 71 °C, with an inset showing the observed 2D‐scattering pattern.

To confirm whether the identification of the N_TB_ phase was correct, we studied compounds **1**–**11** by small angle X‐ray scattering (SAXS) and analysis of the conoscopic figures. As discussed by Zhu et al.,^13^ small‐angle X‐ray scattering can be used to discriminate between the experimentally observed N_TB_ phase and the theoretically predicted splay‐bend nematic (N_SB_).^49^ We note that Gorecka et al. used connoscopy to identify that the N_TB_ phase in chiral dimers is optically positive, that is, the conical angle is less than the magic angle.^36^ Scattering patterns obtained by SAXS for compound **10** and conoscopic figures are given for compound **7** in Figure 4. Analysis of the conoscopic figures of **1**–**11** reveals that in all cases, the N_TB_ phase is uniaxial; insertion of a λ‐plate was used to demonstrate that the twist‐bend phase is optically positive in all cases and therefore, the conical angle is less than the magic angle.^10^, ^36^ In SAXS, the lack of Bragg scattering peaks at small values of *Q* (down to a minimum of *d*=95 Å, *Q*≈0.066) was taken to exclude the possibility of the N_TB_ phase being a splay‐bend modulated structure, as discussed by Zhu et al.^13^

**Figure 5:**
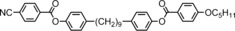
The molecular structure and transition temperatures [°C] of the unsymmetrical polar/apolar dimer reported previously. The SmX‐N_TB_ transition is monotropic and thus presented in parentheses.^50^

**Figure 6:**
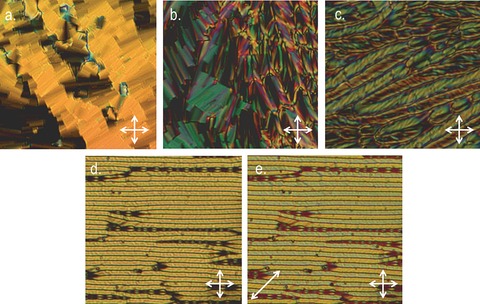
Photomicrographs (×100) of: a) the blocky texture of the twist‐bend phase of **13** at 85.2 °C, b) the blocky texture alongside parabolic defects in the N_TB_ phase of compound **12** at 75.0 °C, c) the rope texture alongside parabolic defects in the N_TB_ of compound **13** at 65.0 °C, d) the well‐aligned rope texture of the N_TB_ phase of compound **12** at 52.5 °C confined in a 5±0.1 μm cell with antiparallel buffed polyimide alignment layers, e) the same conditions as (d) but with a λ‐waveplate inserted.

**Figure 7:**
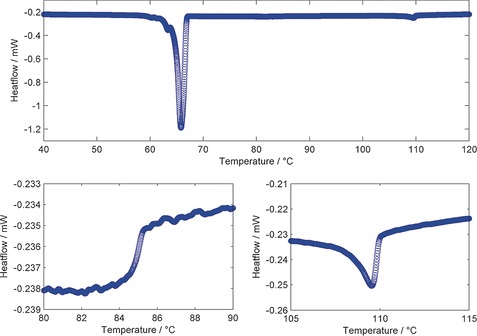
DSC thermogram of compound **13** obtained at 10 °C min^−1^ on the first cooling cycle, with expansions between 80–90 °C showing the nematic to N_TB_ transition and between 105–115 °C showing the nematic to isotropic transition. The large peak at about 66 °C corresponds to recrystallisation of the sample and not a transition to a mesophase.

Recently, we reported a compound (structure in Figure 5) exhibiting the novel phase sequence SmX‐N_TB_‐N,^50^ in which SmX is an unknown smectic mesophase. The syntheses of **12**, **13** and **15** were undertaken primarily to see if further examples of the SmX‐N_TB_ transition could be observed in unsymmetrical bimesogens with one polar and one apolar unit. By studying N_TB_ to smectic phase transitions, it may be possible to gain significant insight into the twist‐bend nematic phase; however, we are aware of only four examples of such phase transitions.^27^, ^39^, ^42^, ^50^ In the case of compound **14**, the incorporation of selenium will hopefully permit future study of the local structure of the N_TB_ phase (as well as the nematic) by resonant small angle X‐ray scattering, TEM and ^77^Se NMR spectroscopy. The transition temperatures and associated enthalpies of four mixed polar/apolar bimesogens are given in Table 3. All four compounds in exhibit nematic and twist‐bend mesophases (identified by POM, DSC and SAXS). Representative photomicrographs showing the characteristic optical textures of the N_TB_ phase are given in Figure 6 and a representative DSC thermogram obtained for compound **13** at 10 °C min^−1^ is given in Figure 7.

**Table 3:** Transition temperatures [°C] and associated enthalpies of transition [kJ mol^−1^] for compounds **12**–**15**. Transitions in parentheses are monotropic, that is, they occur below the melting point of the material in question.

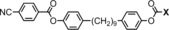								
Compound	X	Cr		N_TB_		N		Iso
**12**	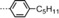	•	88.7 [38.65]	(•	80.7) [0.70]	•	95.1 [0.49]	•
**13**	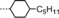	•	91.9 [35.56]	(•	85.2) [0.14]	•	110.0 [1.01]	•
**14**	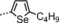	•	71.8 [17.13]	(•	57.7 [0.29]	•	69.6) [0.22]	•
**15**	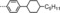	•	125.6 [25.19]	(•	124.5) [0.34]	•	189.6 [0.59]	•

As with compounds **1**–**11**, the associated enthalpies of transition, as determined by DSC at a heat/cool rate of 10 °C min^−1^, are not especially characteristic and do not offer any great insight into the twist‐bend nematic mesophase in this instance. In all cases, the nematic‐isotropic transition was first order, with the nematic‐N_TB_ transition being only weakly first order (see Figure 7).^9^ For compound **15**, the length of one mesogenic unit was increased from two to three rings and, as predicted, this leads to a significant increase in both clearing point and the thermal stability of the N_TB_ phase, highlighting the importance of the aspect ratio, as noted previously.^38^, ^39^, ^43^

**Figure 8:**
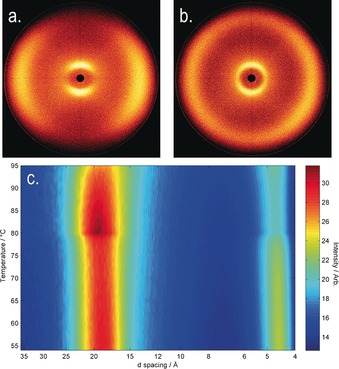
Small angle X‐ray scattering of compound **12**: a) two‐dimensional scattering pattern in the magnetically aligned nematic phase at 94 °C, b) two‐dimensional scattering pattern of the N_TB_ phase at 68 °C (note the loss of alignment), c) contour plot of scattered X‐ray intensity [arb] versus *d*‐spacing [Å] versus temperature [°C].

We elected to study compound **12** by small angle X‐ray scattering over a temperature range of 96–56 °C (1 °C intervals, see Figure 8). As discussed previously, the lack of Bragg scattering peaks in both the nematic and N_TB_ phases at small values of *Q* (down to a minimum of *d*=95 Å, *Q*≈0.066) was taken to exclude the possibility of the N_TB_ phase being a splay‐bend modulated structure. We observed that the small angle peak decreases from an average of 19.3 Å in the nematic phase to 18.8 Å in the N_TB_ phase; this compares with a molecular length of 36.8 Å, obtained at the B3LYP/6–31G(d) level of DFT. The decrease in *d*‐spacing is comparable to that reported previously,^41^ and has been attributed to the molecules tilting away from the helix axis of the N_TB_ phase.^6^ The scattering at wide angles, corresponding to the average lateral molecular separation, presents as a broad peak in the nematic phase. In the twist‐bend nematic phase, this wide‐angle scattering peak becomes noticeably broader, indicating that there is less positional ordering in the N_TB_ than in the nematic phase. Qualitatively, this would present as a reduced order parameter, however the lack of alignment in the N_TB_ phase prevents us from giving quantitative values in this instance.

**Figure 9:**
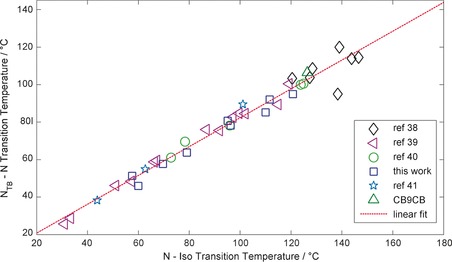
Plot of the nematic to isotropic transition temperature [°C] versus the twist‐bend nematic to nematic transition temperature [°C] for dimers and bimesogens with “two‐ring” mesogenic units and nonamethylene spacer. Data were taken from refs. 38, 39, 40, 41, data for CB9CB are from ref. 11 and “this work” refers to compounds **7**–**15**.

A number of unsymmetrical bimesogens with methylene‐linking groups and nonamethylene (i.e., C_9_) spacers have been prepared and characterised, with all materials found to exhibit the twist‐bend nematic phase. The ability of a wide range of molecular structures to give rise to this mesophase represents a shift in our understanding of the twist‐bend phase from being a curious state of matter that is observed in a sparse number of materials to something universal that can be observed in a (potentially) large number of systems. A “bent” molecular structure is a prerequisite, with the thermal stability of the N_TB_ phase showing a strong dependence on the intermesogen angle.^35^ The twist‐bend phase is most commonly observed in methylene linked dimers with odd spacer parity, notable exceptions being bent‐core systems,^8^ ether‐linked dimers,^27^, ^28^ trimers,^29^, ^30^, ^31^, ^32^ oligomers^33^ and polymers.^34^ Concerning dimers and bimesogens, the largest sub‐group to exhibit the twist‐bend nematic phase are methylene‐linked with a nonamethylene spacer and mesogenic units constructed from two rigid cyclic units, with around 40 materials known in total.^38^, ^39^, ^40^, ^41^, ^51^ We were surprised to find that there is linear relationship (*R* ^2^=0.975) for these materials between the N‐I transition temperature and the N_TB_‐N transition temperature (plot given in Figure 9).

This plot contains polar dimers,^11^, ^38^, ^40^, ^41^ apolar dimers^39^ and bimesogens, yet we find no significant deviation irrespective of polarity. Additionally, the length of terminal alkyl/alkoxy chains employed in apolar dimers has little effect.^39^ Evidently, this plot only includes approximately one third of the twist‐bend materials presently known. If similar plots are made for dimers with 3‐ring mesogenic units or bimesogens with mixed 2‐ and 3‐ ring mesogenic units, then linear relationships also exist, albeit with a different slope. This highlights the importance of the aspect ratio, which we noted previously,^38^, ^39^ in determining the phase stability of dimers. A similar linear relationship also exists for heptamethylene‐linked dimers such as CB7CB and the handful of compounds we reported previously;^17^, ^38^ a plot similar to that in Figure 9 but for the heptamethylene‐linked dimers is given in the Supporting Information of this article (Figure SI‐1).

We expect that a linear relationship will always exist between the NTB‐N and N‐Iso transition temperatures of multiple compounds of homologous structure. Materials with dissimilar structure (e.g., differing aspect ratios, conformer distributions or spacer lengths) are unlikely to yield an analogous linear relationship. These linear fits are only valid for materials with fairly rigid aromatic units and identical linking groups, thereby resulting in architectures that have three well‐defined sub‐units. Conversely, the ether‐linked, mixed ether‐alkyne or mixed ether‐methylene‐linked analogues of CB9CB^35^ do not follow the linear relationship shown in Figure 9. Considering that only a handful of dimers with mixed linking groups are known, it is unclear if analogous linear relationships exist for these materials.

When the linking unit is changed, this will inevitably lead to a change in the distribution of the conformers, and thus the average and also the distribution of the bend angles. As a consequence, the gross topology of the two molecules is no longer the same. Although a linear relationship exists for materials with comparable conformer distributions (i.e., the spacer and linking units are identical), in cases in which conformer distributions are significantly different (e.g., methylene linkers vs. ethers), no linear relationship exists.

If the incidence of the N_TB_ phase is driven by gross topology, an argument that appears to be strongly supported by the present results, we speculate that this phase of matter should manifest on length scales beyond that of the molecule, in a manner analogous to the nematic and smectic phases exhibited by colloidal suspensions of virus particles.^52^, ^53^ However, no such observations have been considered in the literature to date for the N_TB_ phase. These results and their relationship with macro‐ rather than nano‐systems suggest that the topological constraints, rather than the electrostatic interactions, of the molecules in the bulk phase are akin to viewing molecules as molecular grains of matter. Similarly, it is to be expected that supermolecular and supramolecular materials will eventually be found to exhibit the twist‐bend nematic phase, confirming that this state of matter is not confined solely to small molecules.

## Conclusions

In this work, we first expanded the number of materials known to exhibit the twist‐bend nematic mesophase. The discovery of a linear relationship between the nematic to isotropic transition temperature and the N_TB_ to nematic transition temperature is important and shows that although the chemical make‐up of the mesogenic units drives the exact transition temperatures, the relationship between them apparently does not depend on the mesogenic units at all. We find that materials with different spacer lengths, linking groups and aspect ratios still exhibit this linear relationship, albeit with different slopes. The implication is that topology (and therefore presumably the minimisation of free volume^54^) is the driving forces behind the twist‐bend nematic phase. This conclusion is perhaps not a new idea and many will have posited such thoughts in all forms, from pure speculation to detailed theoretical treatments. However, this work provides some of the first experimental evidence supporting the hypothesis that topology dictates the incidence of this phase. For dimeric compounds that exhibit a nematic phase but not the twist‐bend mesophase, it is possible to use knowledge of the slopes of the linear fit for extrapolation of virtual N‐N_TB_ transitions, presenting a complimentary method to extrapolation by the construction of binary phase diagrams that can be used for cross‐checking values. Given that only a handful of oligomeric twist‐bend materials have been reported to date,^29^, ^30^, ^31^, ^32^, ^33^ it is too early to say if this phenomenon also extends into these systems. However, we see no reason why an analogous relationship should not also exist for these oligomeric compounds.

## Experimental Section

All materials were purified by column chromatography over silica gel, followed by filtration to remove insoluble matter and finally recrystallisation. Chemical structure was confirmed by NMR (^1^H, ^13^C{^1^H} and where appropriate ^19^F) and high‐resolution mass spectrometry. Purity was assayed by HPLC using both normal phase (SiO_2_) and reverse phase (C18‐SiO_2_) columns. Polarised optical microscopy (POM) was performed on a Zeiss Axioskop 40Pol microscope. Conoscopic figures were observed using an Olympus BH2 polarising microscope equipped with a Bertrand lens, an Olympus DPlan 100 PO 100x oil immersion objective with a numerical aperture of 1.25. An Olympus λ‐waveplate (part no. 216958) was used to determine the sign of optical anisotropy. Images were captured using a Sony NEX 5R mirrorless digital camera (16.1 megapixels) affixed to the top of the microscope by a custom E‐mount to C‐mount plate. Temperature control during microscopy, both orthoscopic and conoscopic, was afforded by a Mettler FP82HT hotstage controlled by a Mettler FP90 central processor. Differential scanning calorimetry (DSC) was performed on a Mettler DSC822^e^ calibrated before use against indium and zinc standards under an atmosphere of dry nitrogen. Small angle X‐ray scattering was performed on a Bruker D8 Discover using copper Kα radiation (*λ*=0.15418 nm) equipped with a temperature‐controlled, bored graphite rod furnace. Computational chemistry was performed at the B3LYP/6–31G(d) level of DFT in Gaussian G09.^48^ Full experimental details, including chemical characterisation and descriptions of instrumentation used, are available in the Supporting Information of this article.

## Supporting information

As a service to our authors and readers, this journal provides supporting information supplied by the authors. Such materials are peer reviewed and may be re‐organized for online delivery, but are not copy‐edited or typeset. Technical support issues arising from supporting information (other than missing files) should be addressed to the authors.

Supplementary
