# Supplementary material for: Does Topology Dictate the Incidence of the Twist‐Bend Phase? Insights Gained from Novel Unsymmetrical Bimesogens
Source: Chemistry. 2016 Nov 15;22(51):18456–64. doi: 10.1002/chem.201604030 (PMC5217080; doi:10.1002/chem.201604030)
Supplement: Supplementary file 1 — Supplementary [file CHEM-22-18456-s001.pdf]

CHEMISTRY
A European Journal

Supporting Information

Does Topology Dictate the Incidence of the Twist-Bend Phase? Insights Gained from Novel Unsymmetrical Bimesogens

Richard J. Mandle* and John W. Goodby[a]

chem_201604030_sm_miscellaneous_information.pdf

1.1. General Methods.

1-Ethyl-3-(3-dimethylaminopropyl)carbodiimide (EDAC) was purchased from Carbosynth UK, *N,N*-dimethylaminopyridine (DMAP) was purchased from Sigma Aldrich. Selenophene was purchased from TCI chemicals and used without further purification. Solvents were purchased from Fisher Scientific UK and were dried *via* passage over activated alumina prior to use. Nonane-1,9-diylbis(4,1-phenylene) bis(4-cyanobenzoate) (***i1***) was prepared as described previously. [1] The synthesis and chemical characterisation of compounds **1 – 6** is described elsewhere. [2, 3]

Reactions were monitored by thin layer chromatography (TLC) with DCM as the eluent. Silica coated aluminium TLC plates used were purchased from Merck (Kieselgel 60 F-254) and visualised using either UV light (254 nm and 365 nm), or by oxidation with either iodine or aqueous potassium permanganate solution. Yields refer to chromatographically (HPLC) and spectroscopically (^{1}H NMR, ^{13}C{^{1}H} NMR and where appropriate ^{19}F NMR) homogenous material.

NMR spectra were recorded on a JEOL ECS spectrometer operating at 400 MHz (^{1}H), 100.5 MHz (^{13}C{^{1}H} or 376.4 MHz (^{19}F)) as solutions in $CDCl_3$, unless stated otherwise. Mass spectra were recorded on a Bruker micrOTOF MS-Agilent series 1200LC spectrometer. FTIR spectroscopy was performed using a Shimadzu IR Prestige-21 with Specac Golden Gate diamond ATR IR insert. High-performance liquid chromatography was performed on a Shimadzu Prominence modular HPLC system comprising a LC-20A solvent pump, a DGU-20A$_5$ degasser, a SIL-20A autosampler, a CBM-20A communication bus, a CTO-20A column oven, and a SPO-20A dual wavelength UV-vis detector. Purity was assayed using two different stationary phases: reverse-phase chromatography was performed using an Alltech C18 bonded silica column with a 5 µm pore size, an internal diameter of 4.6 mm and a length of 250 mm whilst an Ascentis SI silica column with a 5 µm pore size, an internal diameter of 4.6 mm and a length of 250 mm was used for 'normal' phase chromatography. Assays denoted 'HPLC' are normal phase; those denoted 'RP-HPLC' are reverse-phase. Chromatograms where only one peak was detected are quoted at >99.5% purity.

Polarised optical microscopy was performed on a Zeiss Axioskop 40Pol microscope using a Mettler FP82HT hotstage controlled by a Mettler FP90 central processor. Photomicrographs were captured *via* an InfinityX-21 MP digital camera mounted atop the microscope. Conoscopic figures were observed using an Olympus BH2 polarising microscope equipped with a Bertrand lens, an Olympus DPlan 100 PO 100x oil immersion objective with a numerical aperture of 1.25. An Olympus λ-waveplate (part no. 216958) was used to determine the sign of optical anisotropy. Images were captured using a Sony NEX 5R mirrorless digital camera (16.1 megapixels) affixed to the top of the microscope *via* a custom E-mount to C-mount plate. Temperature control was afforded by a Mettler FP82HT hotstage controlled by a Mettler FP90 central processor.

Differential scanning calorimetry was performed on a Mettler DSC822^e calibrated before use against indium and zinc standards under an atmosphere of dry nitrogen. DSC thermograms were processed in Matlab.

Computational chemistry was performed using the using Gaussian G09 revision d01 on the York Advanced Research Computing Cluster (YARCC) as described in the text. [2]

Small angle X-ray diffraction was performed using a Bruker D8 Discover equipped with a temperature controlled, bored graphite rod furnace, custom built at the University of York. The radiation used was copper Kα (λ = 0.154056 nm) from a 1 µS microfocus source. Diffraction patterns were recorded on a 2048x2048 pixel Bruker VANTEC 500 area detector set at a distance of 121 mm from the sample, allowing simultaneous collection of small angle and wide angle scattering data. Samples were filled into 1mm capillary tubes and aligned with a pair of 1T magnets. Diffraction patterns were collected as a function of temperature and the data processed using Matlab. Raw data are available upon request from the University of York data catalogue.

1.2. Calculated Molecular Dipole Moments

As the dipole moment of a dimer will be heavily dependent on the distribution of conformers we elected to only study the dipole moments of each mesogenic unit used, so **1** is phenyl 4-cyanobenzoate, **7** is phenyl 4-cyano-3-fluorobenzoate and so on. Calculations were performed using the B3LYP hybrid functional and the 6-31G basis set with additional d polarisation (Table **SI1**) or using the B3LYP hybrid functional with Grimme's D3 dispersion and Becke-Johnson damping and the 6-31G basis set with a single diffuse function and additional d polarisation (Table **SI2**). Dipole moments were computed following geometry optimization. 'Angle' refers to the angle between the dipole vector and the mass-inertia axis of the molecular fragment studied.

No.	D_X	D_Y	D_Z	D_{net}	Angle
**1**	4.43	0.69	0.31	4.49	9.74
**2**	2.35	0.98	0.29	2.56	23.48
**3**	4.61	0.76	0.29	4.68	9.93
**4**	0.78	1.31	0.50	1.60	60.71
**5**	2.31	1.08	0.46	2.59	26.91
**6**	3.90	0.99	0.37	4.04	15.12
**7**	5.48	1.43	0.43	5.68	15.24
**8**	2.02	2.20	0.63	3.05	48.57
**9**	2.53	1.08	0.27	2.76	23.78
**10**	5.54	1.27	0.31	5.69	13.29
**11**	3.37	2.02	0.48	3.96	31.71

Table SI1: Dipole moments calculated at the B3LYP /6-31G(d) level of DFT.

No.	D_X	D_Y	D_Z	D_{net}	Angle
**1**	4.62	0.72	0.33	4.68	9.75
**2**	1.91	1.13	0.33	2.24	31.59
**3**	5.00	0.74	0.28	5.06	9.01
**4**	1.21	1.23	0.53	1.80	47.88
**5**	2.93	1.17	0.46	3.19	23.16
**6**	4.55	0.98	0.37	4.67	12.95
**7**	5.86	1.67	0.47	6.11	16.51
**8**	2.66	2.36	0.68	3.62	42.69
**9**	3.28	0.98	0.23	3.43	17.06
**10**	6.00	1.53	0.46	6.21	14.90
**11**	4.13	2.21	0.52	4.72	28.83

Table SI2: Dipole moments calculated at the B3LYP-GD3BJ/6-31+G(d) level of DFT.

1.3. The Relationship between N – Iso and N_{TB} – N Transition Temperatures

1.3.1. Goodness-of-fit Data for Fitting in Figure XXX

SSE	840.3242
R^2	0.9823
DFE	34
Adjusted R^2	0.9817
RMSE	4.9715

Table SI-3: The goodness-of-fit data for the linear fit used in figure **XXX** (see manuscript text). SSE is the sum of squares due to error, DFE is the error degrees of freedom and RMSE is the root mean squared error.

1.3.2. Plot of the N-Iso versus N_{TB} – N Transition Temperatures of Heptamethylene Dimers

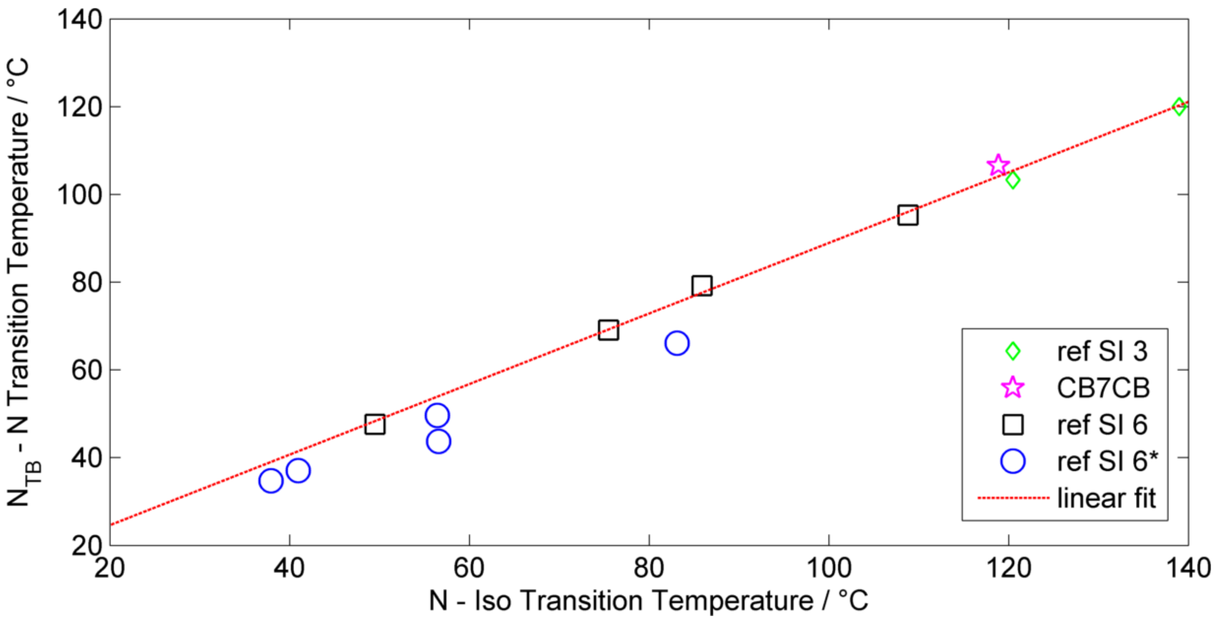

Figure SI-1: Plot of the nematic to isotropic transition temperature (°C) versus the twist-bend nematic to nematic transition temperature (°C) for heptamethylene linked “two-ring” dimers from the literature, with data for CB7CB taken from [5]. Blue circles (denoted with an asterisk) correspond to values obtained by extrapolation from binary mixtures as described in and were not used in fitting due to the presumed errors associated with such extrapolation. [6].

SSE	21.2622
R^2	0.9963
DFE	5
Adjusted R^2	0.9955
RMSE	2.0621

Table SI-4: The goodness-of-fit data for the linear fit used in figure **SI-1**. SSE is the sum of squares due to error, DFE is the error degrees of freedom and RMSE is the root mean squared error. Note, extrapolated data *was not* used during linear fitting.

1.4. General Procedure for the Esterification of Compound *i1* with Benzoic Acids

A solution of compound ***i1*** (50 mg, 0.114 mmol), benzoic acid (0.15 mmol), EDAC (28.7 mg, 0.15 mmol), DMAP (1 mg) and anhydrous DCM (2 ml) in an oven dried micro reaction vial (Supelco) fitted with a mininert valve was stirred under an atmosphere of dry nitrogen gas until complete consumption of ***i1*** as evidenced by TLC (***i1*** Rf_{DCM} = 0.1) and the emergence of a new spot corresponding to the product (Rf_{DCM} = 0.4 – 0.65). The crude material was purified by dry vacuum flash chromatography with DCM as the eluent. The chromatographed material was concentrated, redisolved in DCM and filtered to remove insoluble matter. Recrystalisation of the chromatographed material from ethanol/THF afforded compounds **7-16** in 65-80 % yields.

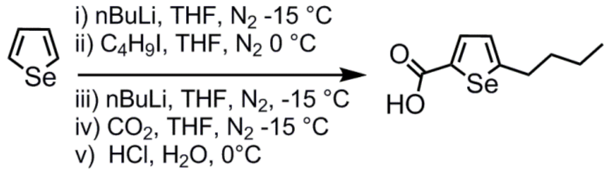

2-Butylselenophene-5-carboxylic acid

Selenophene (100 mg, 0.76 mmol) was dissolved into anhydrous THF (3 ml) and placed into an oven dried 5 ml reaction vial and the solution cooled to – 15°C under an atmosphere of dry nitrogen. N-Butyl lithium (2.5 M in hexanes, 0.4 ml, 1 mmol) was added dropwise, prompting a colour change from pale yellow to orange. Stirring was continued for 15 minutes and the reaction allowed to warm to 0 °C, at which point butyl iodide was added (184 mg, 113 µl 1 mmol). While stirring at 0 °C for 1 hour, the solution changed colour from orange to pale yellow. The reaction was again cooled to – 15 °C and n-butyl lithium (2.5 M in hexanes, 0.5 ml, 1.25 mmol) was added, prompting a colour change to orange. After stirring for 15 minutes, carbon dioxide gas, generated by the action of 2M aqueous hydrochloric acid on calcium carbonate and dried by passage through neat sulphuric acid, was bubbled into the reaction mixture for 15 minutes. The reaction was warmed to 0 °C, stirred for 1 h and then quenched with 2M HCl. The organic layer was separated and retained; the aqueous washed with diethyl ether (3 x 3 ml) and discarded. The combined organic layer was washed with water (1 x 3 ml), brine (1 x 5 ml), dried over $MgSO_4$ and concentrated *in vacuo* to yield a viscous brown oil that slowly crystallised. This oil was purified by column chromatography over silica gel with a gradient of hexanes/ethyl acetate (Rf = 0.1 in 3:1 EtOAc/hexane). The chromatographed material was recrystalised from hexane affording the title compound as a yellow solid.

Yield: 71 mg (40 %)

^{1}H NMR (400 MHz, $CDCl_3$):	0.91 (3H, t, *J* = 7.3 Hz, $-CH_2-C\underline{H}_3-$), 1.38 (2H, sextet, *J* = 7.3 Hz, $-CH_2-C\underline{H}_2-CH_3$), 1.65 (2H, quintet, *J* = 7.3 Hz, $-CH_2-C\underline{H}_2-CH_2-$), 2.85 (2H, t, *J* = 7.3 Hz, $-SeAr-C\underline{H}_2-CH_2-$), 6.93 (1H, dt, J_{H-Se} = 0.7 Hz, J_{H-H} = 3.7 Hz, SeAr$\underline{H}$), 7.82 (1H, d, J_{H-H} = 3.9 Hz, SeAr$\underline{H}$).
$^{13}C\{^1H\}$ NMR (100.5 MHz, $CDCl_3$):	13.72, 22.06, 32.83, 34.37, 127.38, 136.09, 136.86, 162.32, 165.57
FT-IR (v max, cm^{-1}):	763, 916, 1145, 1319, 1375, 1456, 1506, 1541, 1558, 1575, 1653, 2924, 3248 (broad), 3439 (broad)
MS (ESI^+):	233.0073, $C_9H_{13}O_2Se$, M + H {Calcd. for $C_9H_{13}O_2Se$, 233.0075}
	254.9885, $C_9H_{12}NaO_2Se$, M + Na {Calcd. for $C_9H_{12}NaO_2Se$, 254.9895}

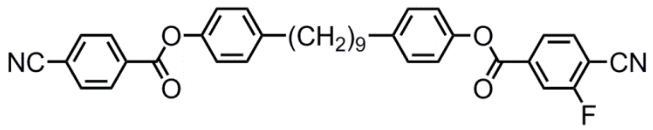

7: 4-(9-(4-((4-cyanobenzoyl)oxy)phenyl)nonyl)phenyl 4-cyano-3-fluorobenzoate

Yield: 48 mg (72 %)

^{1}H NMR (400 MHz, $CDCl_3$): 8.27 (2H, ddd, $J_{H\text{-}H}$ = 1.4 Hz, $J_{H\text{-}H}$ = 2.3 Hz, $J_{H\text{-}H}$ = 8.3 Hz, Ar$\underline{H}$), 8.07 (1H, dd, $J_{H\text{-}H}$ = 1.5 Hz, $J_{H\text{-}F}$ = 7.8 Hz, Ar$\underline{H}$), 8.01 (1H, dd, $J_{H\text{-}H}$ = 1.5 Hz, $J_{H\text{-}F}$ = 9.3 Hz, Ar$\underline{H}$), 7.81 (2H, ddd, $J_{H\text{-}H}$ = 1.4 Hz, $J_{H\text{-}H}$ = 2.3 Hz, $J_{H\text{-}H}$ = 8.3 Hz, Ar$\underline{H}$), 7.78 (1H, dd, $J_{H\text{-}H}$ = 6.1 Hz, $J_{H\text{-}F}$ = 7.8 Hz, Ar$\underline{H}$), 7.24 – 7.17 (5H, m, Ar$\underline{H}$), 7.15 – 7.03 (4H, m, Ar$\underline{H}$), 2.67 – 2.55 (4H, m, Ar-C$\underline{H}_2$-$(CH_2)_7$-C$\underline{H}_2$-Ar), 1.69 – 1.55 (4H, m, Ar-CH_2-C$\underline{H}_2$-$(CH_2)_5$-C$\underline{H}_2$-CH_2-Ar), 1.41 – 1.19 (10H, m, Ar-$(CH_2)_2$-(C$\underline{H}_2$)$_5$-$(CH_2)_2$-Ar)

^{3}C{^{1}H} NMR (100.5 MHz, $CDCl_3$): 163.94, 163.12 (d, $J_{C\text{-}F}$ = 261.1 Hz), 148.51 (d, $J_{C\text{-}F}$ = 23.0 Hz), 136.30 (d, $J_{C\text{-}F}$ = 7.6 Hz), 134.04, 133.73, 132.56, 130.80, 129.72, 129.67, 126.67, 126.29 (d, $J_{C\text{-}F}$ = 3.5 Hz), 121.22, 121.08, 118.11 (d, $J_{C\text{-}F}$ = 21.7 Hz), 118.07, 117.11, 113.26, 35.56, 31.63, 31.61, 29.64, 29.42

^{19}F NMR (376.4 MHz, $CDCl_3$): -104.62 – -104.79 (m, Ar$\underline{F}$)

MS M/Z (ESI+): 611.2318 (calcd. for $C_{37}H_{33}FN_2NaO_4$: 611.2317, M + Na)

Assay (HPLC, peak area): 99.2%

Assay (RP-HPLC, peak area): >99.5%

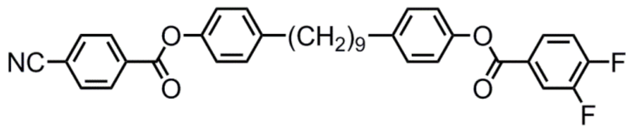

8: 4-(9-(4-((4-cyanobenzoyl)oxy)phenyl)nonyl)phenyl 3,4-difluorobenzoate

Yield:	52 mg (78 %)
^{1}H NMR (400 MHz, $CDCl_3$):	8.29 (2H, ddd, J_{H-H} = 1.4 Hz, J_{H-H} = 2.3 Hz, J_{H-H} = 8.8 Hz, ArH), 8.03 – 7.98 (2H, m, ArH), 7.79 (2H, ddd, J_{H-H} = 1.4 Hz, J_{H-H} = 2.3 Hz, J_{H-H} = 8.8 Hz, ArH), 7.31 – 7.25 (1H, m, ArH), 7.24 – 7.17 (4H, m, ArH), 7.10 (2H, ddd, J_{H-H} = 1.5 Hz, J_{H-H} = 2.7 Hz, J_{H-H} = 8.5 Hz, ArH), 7.07 (2H, ddd, J_{H-H} = 1.8 Hz, J_{H-H} = 2.7 Hz, J_{H-H} = 8.5 Hz, ArH), 2.65 – 2.54 (4H, m [t + t], Ar-CH_2-$(CH_2)_7$-CH_2Ar), 1.68 – 1.54 (4H, m, Ar-CH_2-CH_2-$(CH_2)_5$-CH_2-CH_2-Ar), 1.48 – 1.15 (10H, m, Ar-$(CH_2)_2$-(CH_2)$_5$-$(CH_2)_2$-Ar
^{13}C{^{1}H} NMR (100.5 MHz, $CDCl_3$):	163.93, 163.53 (d, J_{C-F} = 2.3 Hz), 154.15 (dd, J_{C-F} = 12.7 Hz, J_{C-F} = 257.2, Hz), 150.36 (dd, J_{C-F} = 13.1 Hz, J_{C-F} = 250.4 Hz), 148.68, 148.58, 141.27, 141.06, 133.73, 132.55, 130.79, 129.67, 129.60, 127.36 (dd, J_{C-F} = 3.6 Hz, J_{C-F} = 7.3 Hz), 126.88 (dd, J_{C-F} = 4.0 Hz, J_{C-F} = 5.7 Hz), 121.30, 121.21, 119.68 (dd, J_{C-F} = 2.1 Hz, J_{C-F} = 18.4 Hz), 118.07, 117.85, 117.67, 117.09, 35.55, 31.62, 29.65, 29.63, 29.42.
^{19}F NMR (376.4 MHz, $CDCl_3$):	-128.94 (1F, tdd, J_{H-F} = 4.6 Hz, J_{F-F} = 94 Hz, J_{C-F} = 10.1Hz, ArF), -135.92 (ddd, J_{H-F} = 7.5 Hz, J_{F-F} = 9.4 Hz, J_{C-F} = 17.8 Hz, ArF)
MS M/Z (ESI+):	604.2256 (calcd. for $C_{36}H_{33}F_2NNaO_4$: 604.2270, M + Na)
Assay (HPLC, peak area):	99.4%
Assay (RP-HPLC, peak area):	>99.5%

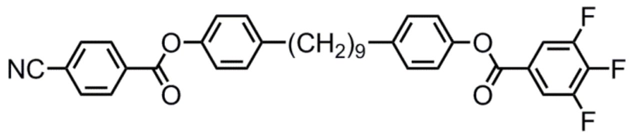

9: 4-(9-(4-((4-cyanobenzoyl)oxy)phenyl)nonyl)phenyl 3,4,5-trifluorobenzoate

Yield:	51 mg (76 %)
^{1}H NMR (400 MHz, $CDCl_3$):	8.28 (2H, ddd, J_{H-H} = 2.4 Hz, J_{H-H} = 1.8 Hz, J_{H-H} = 8.8, Ar$\underline{H}$), 7.87 – 7.74 (4H, m, Ar$\underline{H}$), 7.24 – 7.18 (4H, m, Ar$\underline{H}$), 7.13 – 7.03 (4H, m, Ar$\underline{H}$), 2.91 – 2.41 (4H, m [2 x t], Ar-C$\underline{H}_2$-$(CH_2)_7$-C$\underline{H}_2$-Ar), 1.70 – 1.56 (4H, m, Ar-CH_2-C$\underline{H}_2$-$(CH_2)_5$-C$\underline{H}_2$-CH_2-Ar), 1.42 – 1.17 (10H, m, Ar-$(CH_2)_2$-(C$\underline{H}_2$)$_5$-$(CH_2)_2$-Ar).
^{13}C{^{1}H} NMR (100.5 MHz, $CDCl_3$):	163.93, 162.79, 151.29 (dd, J_{C-F} = 7.0 Hz, J_{C-F} = 245.8 Hz), 149.97 (d, J_{C-F} = 3.1 Hz), 148.55 (d, J_{C-F} = 7.7 Hz), 141.29, 133.73, 132.55, 130.79, 129.66, 121.19 (d, J_{C-F} = 5.0 Hz), 118.07, 117.09, 115.06, 115.00, 114.95 (dd, J_{C-F} = 16.6, 6.3 Hz)., 35.55, 31.61, 29.65, 29.63, 29.42.
^{19}F NMR (376.4 MHz, $CDCl_3$):	-132.08 (2F, dd, J_{H-F} = 7.1 Hz, J_{F-F} = 20.1 Hz), -151.38 (1F, tt, J_{H-F} = 6.4 Hz, J_{F-F} = 20.1 Hz).
MS M/Z (ESI+):	620.2023 (calcd. for $C_{36}H_{30}F_3NNaO_4$: 620.2019, M + Na)
Assay (HPLC, peak area):	>99.5%
Assay (RP-HPLC, peak area):	99.1%

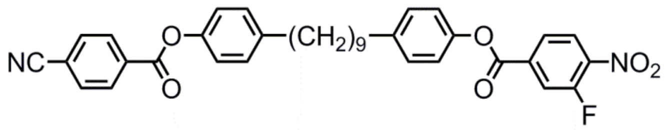

10: 4-(9-(4-((4-cyanobenzoyl)oxy)phenyl)nonyl)phenyl 3-fluoro-4-nitrobenzoate

Yield: 44 mg (65%)

^{1}H NMR (400 MHz, $CDCl_3$): 8.28 (2H, ddd, J_{H-H} = 1.8 Hz, J_{H-H} = 2.8 Hz, J_{H-H} = 8.3 Hz ,ArH), 8.20 – 8.01 (3H, m, ArH), 7.86 – 7.76 (2H, m, ArH), 7.24 – 7.18 (4H, m, ArH), 7.10 (2H, ddd, J_{H-H} = 8.7 Hz, J_{H-H} = 3.0 Hz, J_{H-H} = 1.8 Hz, ArH), 7.08 (2H, ddd, J_{H-H} = 8.7 Hz, J_{H-H} = 3.0 Hz, J_{H-H} = 1.8 Hz, ArH), 2.68 – 2.51 (4H, m, Ar-C$\underline{H}_2$-$(CH_2)_7$-C$\underline{H}_2$-Ar), 1.73 – 1.56 (4H, m, Ar-CH_2-C$\underline{H}_2$-$(CH_2)_5$-C$\underline{H}_2$-CH_2-Ar), 1.42 – 1.16 (10H, m, Ar-$(CH_2)_2$-(C$\underline{H}_2$)$_5$-$(CH_2)_2$-Ar)

^{13}C{^{1}H} NMR (100.5 MHz, $CDCl_3$): 163.93, 162.56 (d, J_{C-F} = 2.3 Hz), 155.31 (d, J_{C-F} = 266.3 Hz) 148.59, 148.40, 141.54, 141.26, 140.52 (d, J_{C-F} = 8.2 Hz), 136.39 (d, J_{C-F} = 7.6 Hz)133.71, 132.56, 130.79, 129.73, 129.66, 126.53 (d, J_{C-F} = 2.1 Hz), 126.22 (d, J_{C-F} = 4.6 Hz) 121.22, 121.06, 120.50, 120.28, 118.07, 117.10, 35.55, 31.62, 31.60, 29.63, 29.42.

^{19}F NMR (376.4 MHz, $CDCl_3$): -116.01 (dd, J_{H-F} = 6.9 Hz, J_{F-F} = 10.4 Hz).

MS M/Z (ESI+): 631.2186 (calcd. for $C_{36}H_{33}FN_2NaO_6$: 631.2215, M + Na)

Assay (HPLC, peak area): 99.2%

Assay (RP-HPLC, peak area): 99.0%

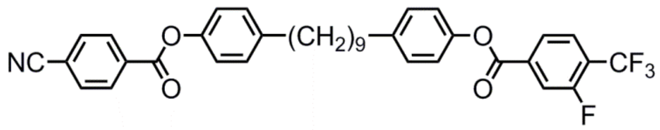

11: 4-(9-(4-((4-cyanobenzoyl)oxy)phenyl)nonyl)phenyl 3-fluoro-4-trifluoromethylbenzoate

Yield: 53 mg (79%)

^{1}H NMR (400 MHz, $CDCl_3$): 8.28 (2H, ddd, J_{H-H} = 1.8 Hz, J_{H-H} = 2.4 Hz, J_{H-H} = 8.4 Hz, Ar$\underline{H}$), 8.06 (1H, d, J_{H-F} = 8.2 Ar$\underline{H}$), 7.99 (1H, d, J_{H-F} = 10.6 Hz, Ar$\underline{H}$), 7.80 (2H, ddd, J_{H-H} = 1.9 Hz, J_{H-H} = 2.4 Hz, J_{H-H} = 8.4 Hz, Ar$\underline{H}$), 7.75 (1 H, dd, J 8.3, 6.8), 7.22 (2H, ddd, J_{H-H} = 1.9 Hz, J_{H-H} = 2.4 Hz, J_{H-H} = 8.4 Hz, Ar$\underline{H}$), 7.09 (2H, ddd, J_{H-H} = 1.8 Hz, J_{H-H} = 2.4 Hz, J_{H-H} = 8.4 Hz, Ar$\underline{H}$), 2.67 – 2.55 (4H, m, Ar-C$\underline{H}_2$-(CH_2)$_7$-C$\underline{H}_2$-Ar), 1.69 – 1.56 (4H, m, Ar-CH_2-C$\underline{H}_2$-(CH_2)$_5$-C$\underline{H}_2$-CH_2-Ar), 1.38 – 1.16 (10H, m, Ar-(CH_2)$_2$-(C$\underline{H}_2$)$_5$-(CH_2)$_2$-Ar)

^{13}C{^{1}H} NMR (100.5 MHz, $CDCl_3$): 163.93, 163.25 (d, J_{C-F} = 2.1 Hz), 159.63 (d, J_{C-F} = 222.6 Hz) 148.55 (d, J_{C-F} = 6.9 Hz), 141.30 (d, J_{C-F} = 6.1 Hz), 135.57 (d, J_{C-F} = 7.5 Hz) 133.72, 132.55, 130.79, 129.67, 127.78 (d, J_{C-F} = 4.6 Hz), 125.85 (d, J_{C-F} = 4.2 Hz), 121.21, 121.17, 118.79, 118.56, 118.07, 117.09, 35.55, 31.62, 29.66, 29.63, 29.42.

^{19}F NMR (376.4 MHz, $CDCl_3$): -61.75 (3F, d, J_{H-F} = 16.5 Hz, J_{C-F} = 272.9 Hz, Ar-C$\underline{F}_3$), -112.54 – -112.77 (1F, m, Ar$\underline{F}$).

MS M/Z (ESI+): 654.2238 ($C_{37}H_{33}F_4NNaO_4$: 654.2238, M + Na)

Assay (HPLC, peak area): 99.3%

Assay (RP-HPLC, peak area): >99.5%

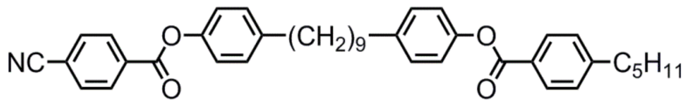

12: 4-(9-(4-((4-cyanobenzoyl)oxy)phenyl)nonyl)phenyl 4-pentylbenzoate

Yield:	51 mg (76%)
^{1}H NMR (400 MHz, $CDCl_3$):	8.28 (2H, ddd, J_{H-H} = 1.5 Hz, J_{H-H} = 2.1 Hz, J_{H-H} = 8.5 Hz, Ar$\underline{H}$), 8.09 (2H, ddd, J_{H-H} = 1.5 Hz, J_{H-H} = 2.1 Hz, J_{H-H} = 8.5 Hz, Ar$\underline{H}$), 7.79 (2H, ddd, J_{H-H} = 1.5 Hz, J_{H-H} = 2.1 Hz, J_{H-H} = 8.5 Hz, Ar$\underline{H}$), 7.29 (2H, ddd, J_{H-H} = 1.5 Hz, J_{H-H} = 2.1 Hz, J_{H-H} = 8.5 Hz, Ar$\underline{H}$), 7.24 – 7.17 (4H, m, Ar$\underline{H}$), 7.13 – 7.05 (4H, m, Ar$\underline{H}$), 2.68 (2H, t, J_{H-H} 7.3, Ar-C$\underline{H}_2$-$(CH_2)_3$-CH_3), 2.64 – 2.56 (4H, m, Ar-C$\underline{H}_2$-$(CH_2)_7$-C$\underline{H}_2$-Ar), 1.72 – 1.44 (6H, m, Ar-CH_2-C$\underline{H}_2$-$(CH_2)_2$-CH_3 + Ar-CH_2-C$\underline{H}_2$-$(CH_2)_5$-C$\underline{H}_2$-CH_2-Ar), 1.42 – 1.22 (14H, m, Ar-$(CH_2)_2$-C$\underline{H}_2$-C$\underline{H}_2$-CH_3 + Ar-$(CH_2)_2$-(C$\underline{H}_2$)$_5$-(CH_2)-Ar), 0.88 (3H, t, J_{H-H} = 6.9 Hz, Ar-$(CH_2)_4$-C$\underline{H}_3$).
^{13}C{^{1}H} NMR (100.5 MHz, $CDCl_3$):	165.62, 163.92, 149.45, 149.05, 148.56, 141.28, 140.57, 133.73, 132.54, 130.79, 130.38, 129.67, 129.48, 128.80, 127.25, 121.54, 121.20, 118.08, 117.06, 36.22, 35.55, 31.66, 31.61, 31.59, 31.02, 29.63, 29.42, 29.40, 22.68, 14.19.
MS M/Z (ESI+):	638.3216 (calcd. for $C_{41}H_{45}NNaO_4$: 638.3241, M + Na)
Assay (HPLC, peak area):	>99.5%
Assay (RP-HPLC, peak area):	>99.5%

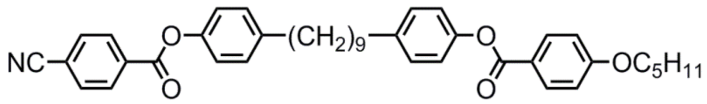

13: 4-(9-(4-((4-cyanobenzoyl)oxy)phenyl)nonyl)phenyl 4-(pentyloxy)benzoate

Yield:	52 mg (73%)
^{1}H NMR (400 MHz, $CDCl_3$):	8.29 (2H, ddd, J_{H-H} = 1.8 Hz, J_{H-H} = 2.5 Hz, J_{H-H} = 8.9 Hz, ArH), 8.10 (2H, ddd, J_{H-H} = 1.8 Hz, J_{H-H} = 2.5 Hz, J_{H-H} = 8.9 Hz, ArH), 7.79 (2H, ddd, J_{H-H} = 1.8 Hz, J_{H-H} = 2.4 Hz, J_{H-H} = 8.5 Hz, ArH), 7.21 (2H, ddd, J_{H-H} = 1.8 Hz, J_{H-H} = 2.4 Hz, J_{H-H} = 8.5 Hz, ArH), 7.16 – 7.00 (4H, m, ArH), 6.94 29 (2H, ddd, J_{H-H} = 1.8 Hz, J_{H-H} = 2.5 Hz, J_{H-H} = 8.9 Hz, ArH), 4.02 (2H, t, J_{H-H} = 6.6 Hz, ArO-CH_2-CH_2-), 2.73 – 2.56 (4H, m, Ar-CH_2-$(CH_2)_7$-CH_2-Ar),1.87 – 1.70 (2H, m, Ar-CH_2-CH_2-$(CH_2)_2$-CH_3), 1.70 – 1.52 (4H, m, Ar-CH_2-CH_2-$(CH_2)_5$-CH_2-CH_2-Ar), 1.51 – 1.19 (14H, m, ArO-$(CH_2)_2$-CH_2-CH_2-CH_3 + Ar-$(CH_2)_2$-$(CH_2)_5$-(CH_2)-Ar), 0.93 (3H, t, J_{H-H} = 6.9 Hz, ArO-$(CH_2)_4$-CH_3).
^{13}C{^{1}H} NMR (100.5 MHz, $CDCl_3$):	165.33, 163.93, 163.62, 149.10, 148.57, 141.28, 140.48, 133.73, 132.55, 132.40, 130.79, 129.67, 129.45, 121.84, 121.59, 121.20, 118.09, 117.06, 114.41, 68.46, 35.55, 31.66, 31.62, 29.63, 29.42, 29.40, 28.97, 28.31, 22.62, 14.20.
MS M/Z (ESI+):	654.3186 (calcd. for $C_{41}H_{45}NNaO_5$: 654.3190, M + Na)
Assay (HPLC, peak area):	99.2%
Assay (RP-HPLC, peak area):	>99.5%

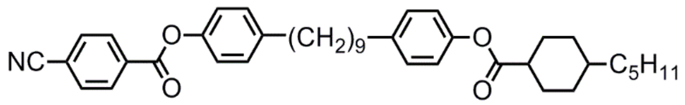

14: 4-(9-(4-((4-pentylcyclohexanecarbonyl)oxy)phenyl)nonyl)phenyl 4-cyanobenzoate

Yield: 44 mg (65%)

^{1}H NMR (400 MHz, $CDCl_3$): 8.28 (2H, ddd, J_{H-H} = 1.8 Hz, J_{H-H} = 2.3 Hz, J_{H-H} = 8.4 Hz, ArH), 7.21 (2H, ddd, J_{H-H} = 1.8 Hz, J_{H-H} = 2.3 Hz, J_{H-H} = 8.4 Hz, ArH), 7.15 (2H, ddd, J_{H-H} = 1.8 Hz, J_{H-H} = 2.3 Hz, J_{H-H} = 8.4 Hz, ArH), 7.09 (2H, ddd, J_{H-H} = 1.8 Hz, J_{H-H} = 2.3 Hz, J_{H-H} = 8.4 Hz, ArH), 6.93 (2H, ddd, J_{H-H} = 1.8 Hz, J_{H-H} = 2.3 Hz, J_{H-H} = 8.4 Hz, ArH), 2.63 – 2.58 (4H, m, Ar-CH_2-$(CH_2)_7$-CH_2-Ar), 2.58 – 2.53 (2H, t, J_{H-H} = 7.1 Hz, Cy-CH_2-$(CH_2)_3$-CH_3), 2.44 (1H, tt, J_{H-H} = 3.4 Hz, J_{H-H} = 12.2 Hz, CyH), 2.16 – 2.03 (2H, m, CyH), 1.91 – 1.78 (2H, m, CyH), 1.69 – 1.43 (7H, m, CyH), 1.38 – 1.13 (18H, m, -CH_2-), 0.95 (2H, dQuartet, J_{H-H} = 3.5 Hz, J_{H-H} = 13.7 Hz, CyH), 0.87 (3H, t, J_{H-H} = 7.1 Hz, Cy-$(CH_2)_4$-CH_3).

^{13}C{^{1}H} NMR (100.5 MHz, $CDCl_3$): 175.12, 163.92, 148.91, 148.57, 141.28, 140.39, 133.74, 132.54, 130.79, 129.67, 129.37, 121.33, 121.20, 118.07, 117.08, 43.82, 37.33, 37.09, 35.54, 35.51, 32.46, 32.33, 31.64, 31.61, 29.63, 29.41, 29.20, 26.70, 22.86, 14.29.

MS M/Z (ESI+): 622.3839 (calcd. for $C_{41}H_{52}NO_4$, M + H)
644.3680 (calcd. for $C_{41}H_{51}NNaO_4$: 644.3710, M + Na)

Assay (HPLC, peak area): >99.5%

Assay (RP-HPLC, peak area): >99.5%

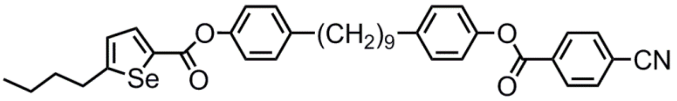

15: 4-(9-(4-((4-Cyanobenzoyl)oxy)phenyl)nonyl)phenyl 5-butylselenophene-2-carboxylate

Quantities: 2-butylselenophene-5-carboxylic acid (70 mg, 302 µmol), 4-(9-(4-hydroxyphenyl)nonyl)phenyl 4-cyanobenzoate (131 mg, 297 µmol), EDAC (191 mg, 1 mmol) and DMAP (1 mg) dry dichloromethane (3 ml). The experimental procedure was in keeping with the general method. Purification was by chromatography with 1:1 DCM/hexanes as the eluent (Rf_{DCM} = 0.8) followed by filtration through a 0.2 µm microfilter (cellulose) before recrystalisation from 1:10 DCM/hexanes to afford the title compound as a white powder.

Yield:	155 mg (79%)
^{1}H NMR (400 MHz, $CDCl_3$):	0.94 (3H, t, *J* = 7.3 Hz, -CH_2-C$\underline{H}_3$), 1.18 – 1.36 (10H, m, -CH_2-(C$\underline{H}_2$)$_5$-CH_2-), 1.40 (2H, sextet, *J* = 7.3 Hz, -CH_2-C$\underline{H}_2$-CH_3), 1.56 – 1.63 (4H, m, Ar-CH_2-C$\underline{H}_2$-(CH_2)$_5$-C$\underline{H}_2$-CH_2-Ar), 1.63 – 1.73 (2H, m, SeAr-CH_2-C$\underline{H}_2$-CH_2-CH_3), 2.58 (2H, t, J_{H-H} = 7.3 Hz, J_{H-Se} = 12.2 Hz, (Se)Ar-C$\underline{H}_2$-CH_2-), 2.61 (2H, t, *J* = 7.3 Hz, Ar-C$\underline{H}_2$-CH_2-), 2.90 (2H, t, *J* = 7.3 Hz, SeAr-C$\underline{H}_2$-CH_2-), 7.05 (1H, dt, J_{H-Se} = 1.0 Hz, J_{H-H} = 3.7 Hz, SeAr$\underline{H}$), 7.07 (2H, ddd, *J* = 1.8 Hz, *J* = 2.8 Hz, *J* = 8.7 Hz, Ar$\underline{H}$), 7.10 (2H, ddd, *J* = 1.8 Hz, *J* = 2.8 Hz, *J* = 8.7 Hz, Ar$\underline{H}$), 7.18 (2H, ddd, *J* = 2.3 Hz, *J* = 2.8 Hz, *J* = 8.7 Hz, Ar$\underline{H}$), 7.22 (2H, ddd, *J* = 2.3 Hz, *J* = 2.8 Hz, *J* = 8.7 Hz, Ar$\underline{H}$), 7.97 (2H, ddd, *J* = 1.4 Hz, *J* = 1.8 Hz, *J* = 8.7 Hz, Ar$\underline{H}$), 8.01 (1H, d, *J* = 3.7 Hz, SeAr$\underline{H}$), 8.28 (2H, ddd, *J* = 1.4 Hz, *J* = 1.8 Hz, *J* = 8.7 Hz, Ar$\underline{H}$)
^{13}C{^{1}H} NMR (100.5 MHz, $CDCl_3$):	13.78, 22.11, 29.21, 29.43, 31.42, 31.44, 32.94, 34.45, 35.35, 116.86, 117.89, 121.00, 121.28, 127.70, 129.24, 129.48, 130.60, 132.35, 133.53, 134.89, 137.40, 140.42, 141.08, 148.36, 148.65, 162.06, 163.73, 163.81,
FT-IR (ν max, cm^{-1}):	686, 736, 761, 817, 862, 887, 991, 1018, 1047, 1074, 1165, 1174, 1190, 1259, 1465, 1506, 1710, 1737, 2231, 2852, 2924
MS (ESI$^+$):	678.2097 (Calcd. for $C_{38}H_{41}NNaO_4Se$: 678.2093, M + Na)
Assay (HPLC, peak area):	>99.5%
Assay (RP-HPLC, peak area):	>99.5%

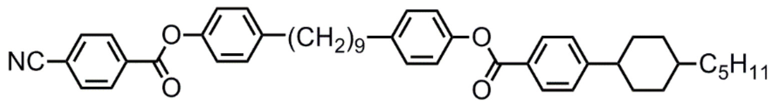

16: 4-(9-(4-((4-cyanobenzoyl)oxy)phenyl)nonyl)phenyl 4-(4-pentylcyclohexyl)benzoate

Yield: 51 mg (71%)

^{1}H NMR (400 MHz, $CDCl_3$): 8.28 (2H, d, J_{H-H} = 8.3 Hz, Ar<u>H</u>), 8.09 (2H, d, J_{H-H} = 8.3 Hz, Ar<u>H</u>), 7.79 (2H, d, J_{H-H} = 8.5 Hz, Ar<u>H</u>), 7.32 (2H, d, J_{H-H} = 8.3 Hz, Ar<u>H</u>), 7.22 (2H, d, J_{H-H} = 8.5 Hz, Ar<u>H</u>), 7.19 (2H, d, J_{H-H} = 8.3 Hz, Ar<u>H</u>), 7.09 (2H, d, J_{H-H} = 8.3 Hz, Ar<u>H</u>), 7.07 (2H, d, J_{H-H} = 8.3 Hz, Ar<u>H</u>), 2.66 – 2.47 (5H, m, Ar-C<u>H</u>$_2$-(CH_2)$_7$-C<u>H</u>$_2$-Ar + Cy<u>H</u>), 2.02 – 1.78 (4H, m, Cy<u>H</u>), 1.67 – 1.39 (8H, m, Cy<u>H</u> + Cy-CH_2-C<u>H</u>$_2$), 1.39 – 1.16 (19H, m, -C<u>H</u>$_2$- + Cy<u>H</u>), 1.13 – 0.97 (2H, m, Cy<u>H</u>), 0.88 (3H, t, J_{H-H} = 7.0 Hz, Ar<u>H</u>)

^{13}C{^{1}H} NMR (100.5 MHz, $CDCl_3$): 165.60, 163.93, 154.28, 149.07, 148.58, 141.29, 140.57, 133.75, 132.55, 130.80, 130.46, 129.68, 129.48, 127.41, 127.26, 121.55, 121.21, 118.09, 117.08, 45.02, 37.50, 37.42, 35.56, 34.23, 33.60, 32.37, 31.66, 31.62, 29.64, 29.41, 26.81, 22.90, 14.31.

MS M/Z (ESI+): 698.4183 (calcd. for $C_{47}H_{55}NO_4$: 698.4204, M + H)

Assay (HPLC, peak area): 99.1%

Assay (RP-HPLC, peak area): 99.3%

1.5. Supplemental NMR spectra

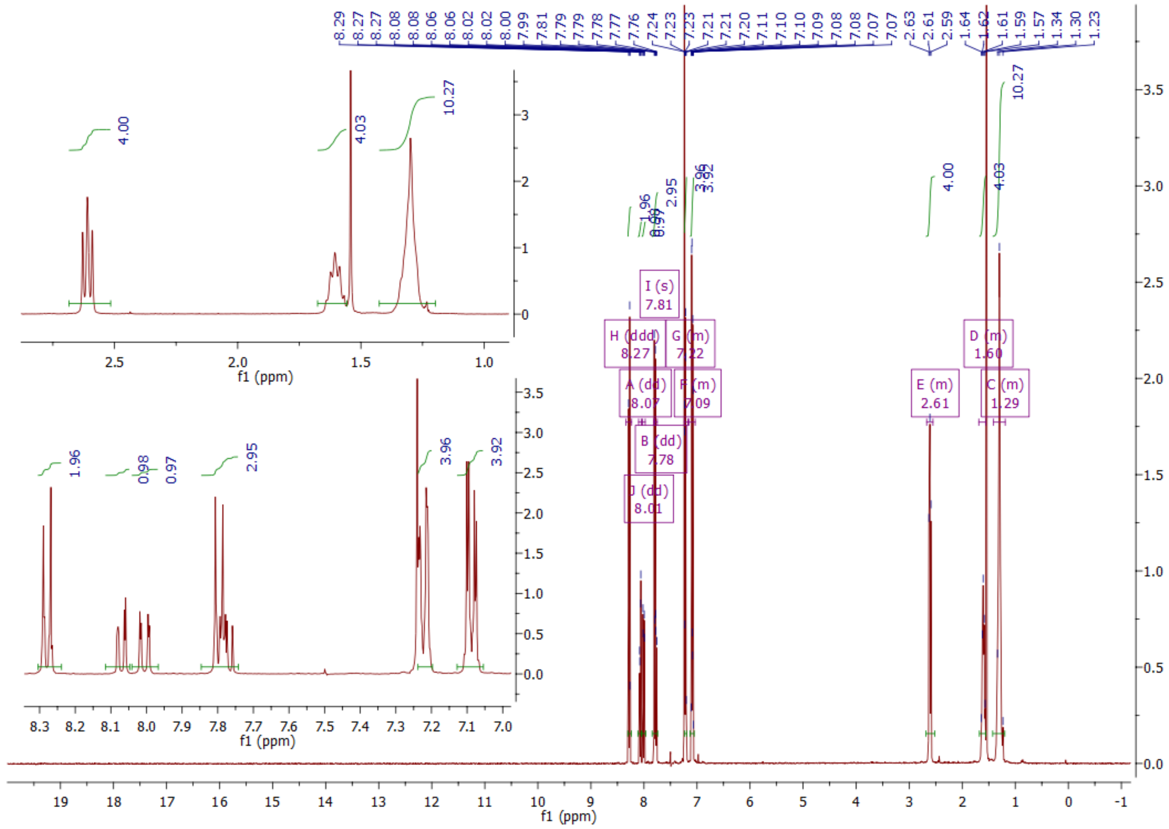

Figure SI-2: ^{1}H (400 MHz, $CDCl_3$) NMR spectra of compound **7**

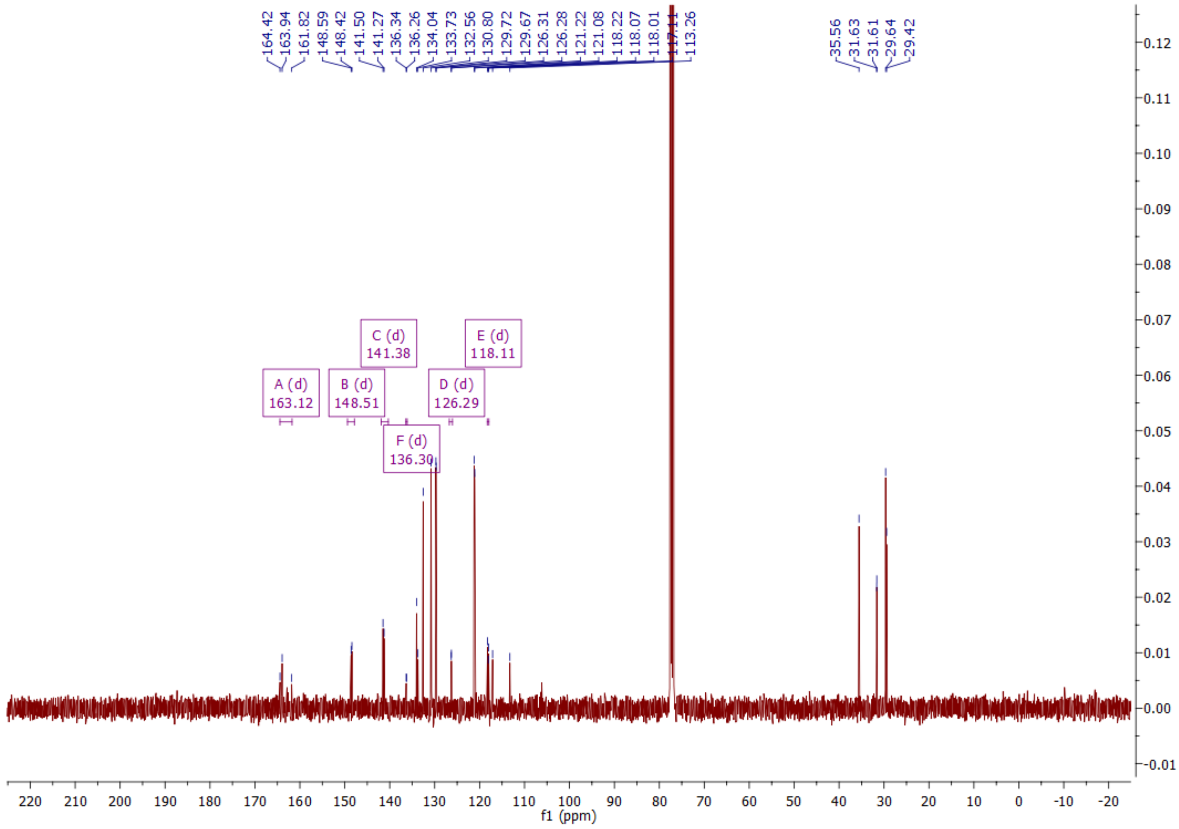

Figure SI-3: $^{13}C\{^{1}H\}$ (100.5 MHz, $CDCl_3$) NMR spectra of compound **7**

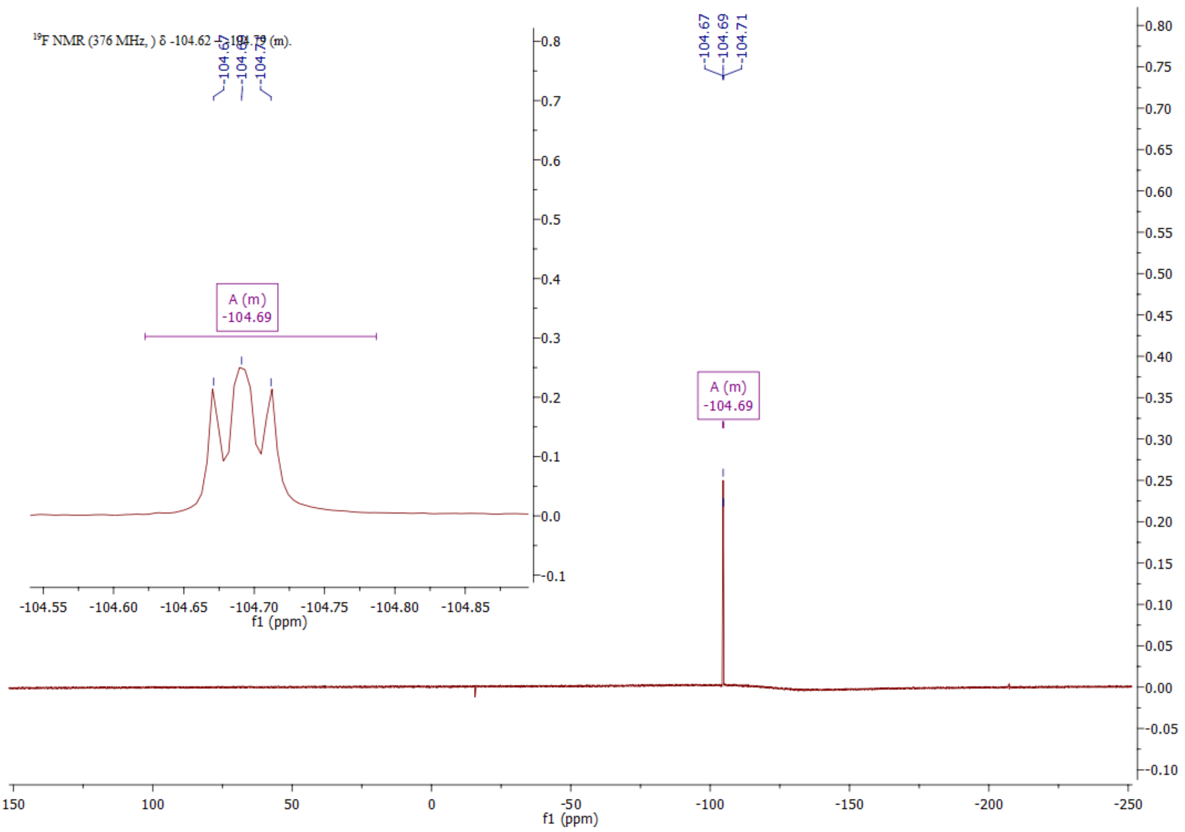

Figure SI-4: ^{19}F (376.4 MHz, $CDCl_3$) NMR spectra of compound **7**

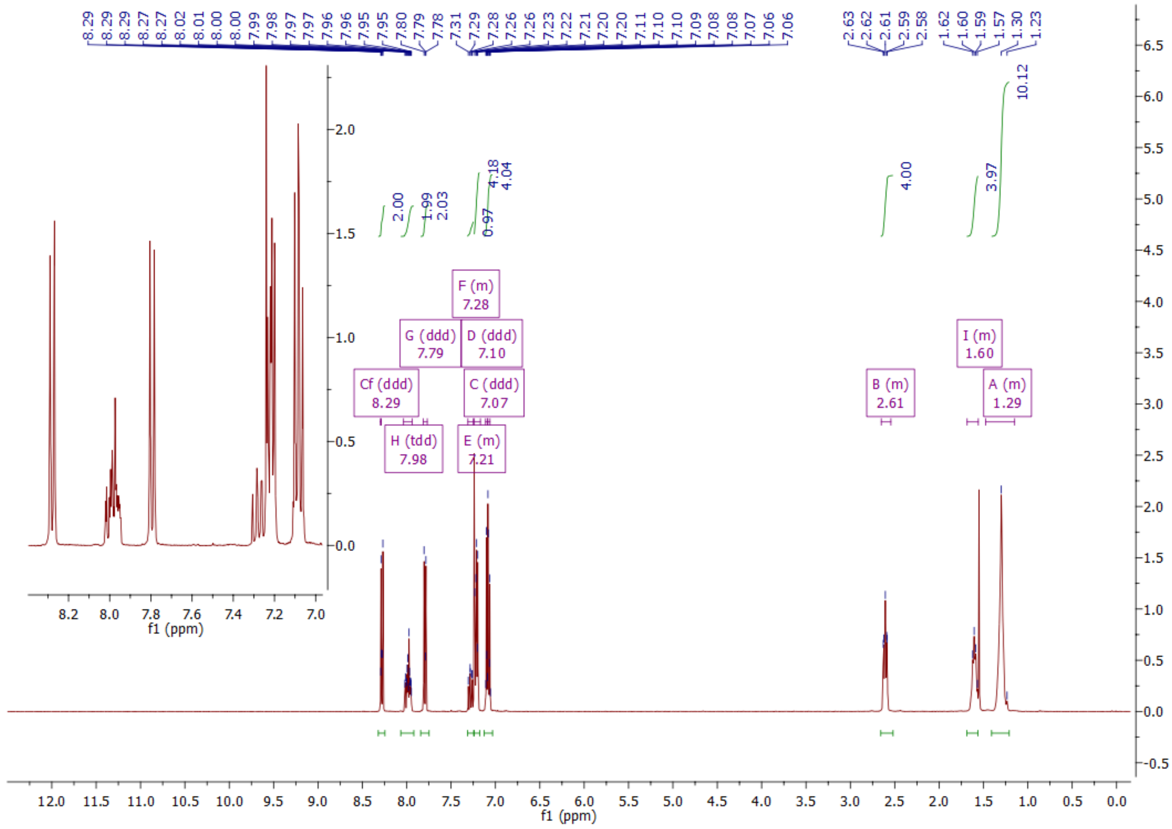

Figure SI-5: ^{1}H (400 MHz, $CDCl_3$) NMR spectra of compound **8**

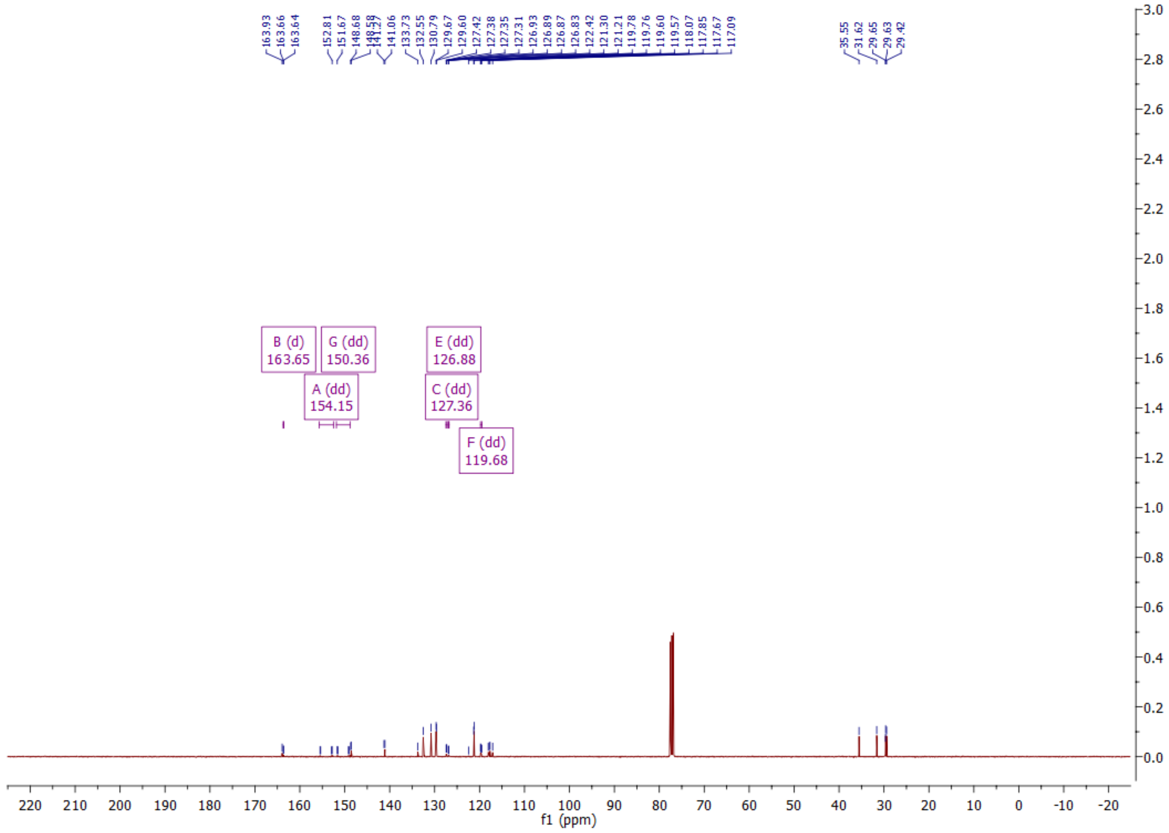

Figure SI-6: $^{13}C\{^1H\}$ (100.5 MHz, $CDCl_3$) NMR spectra of compound **8**

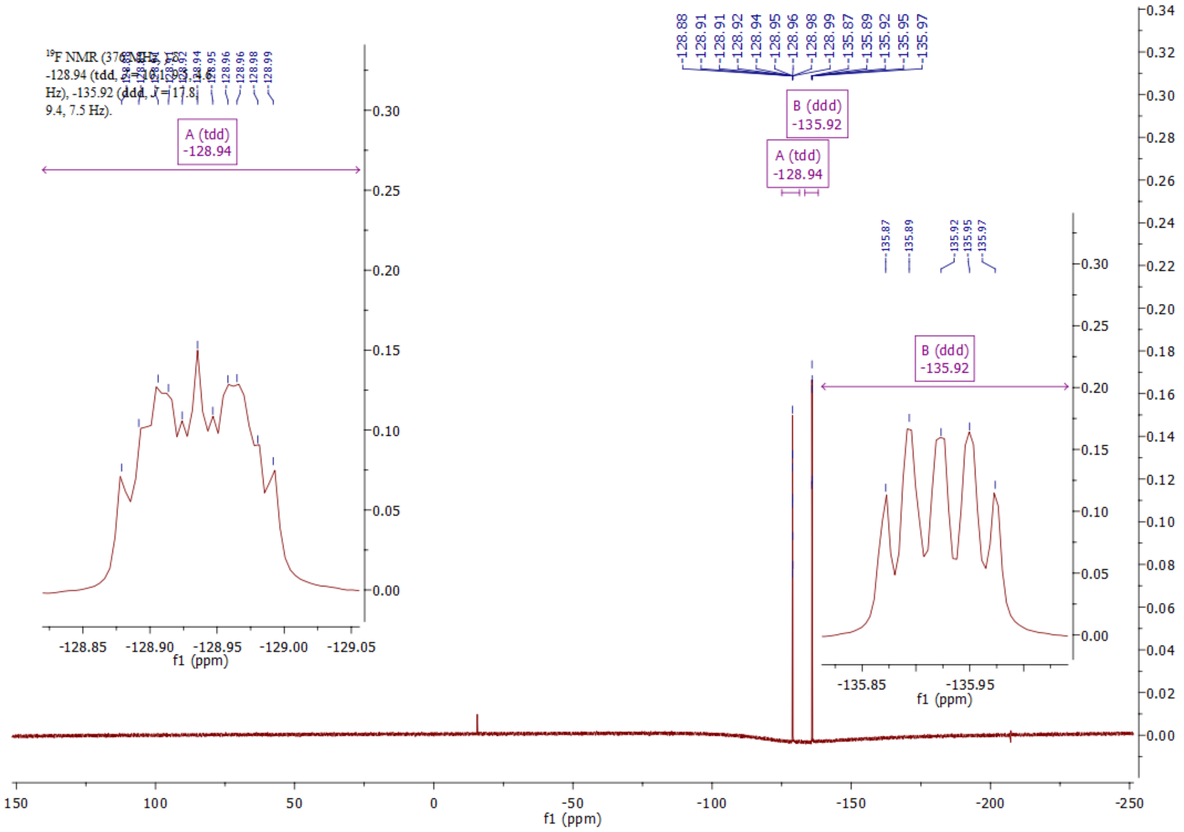

Figure SI-7: ^{19}F (376.4 MHz, $CDCl_3$) NMR spectra of compound **8**

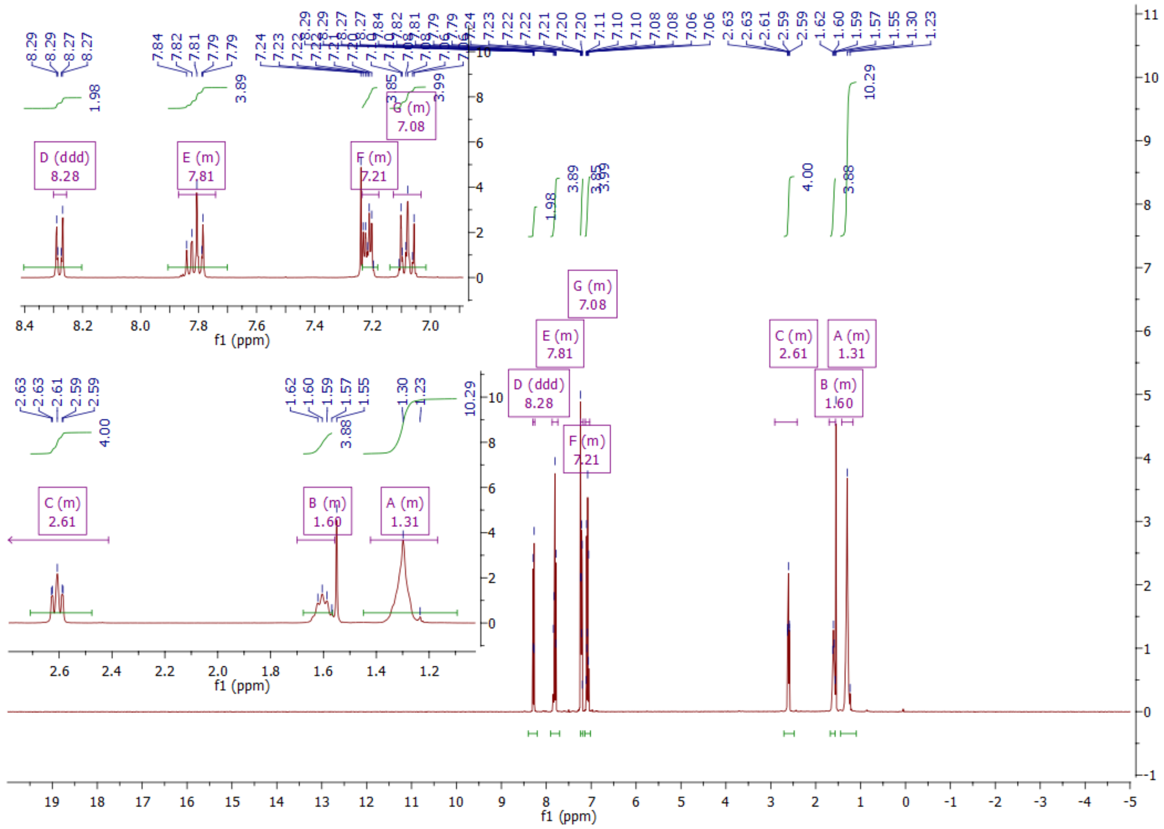

Figure SI-8: ^{1}H (400 MHz, $CDCl_3$) NMR spectra of compound **9**

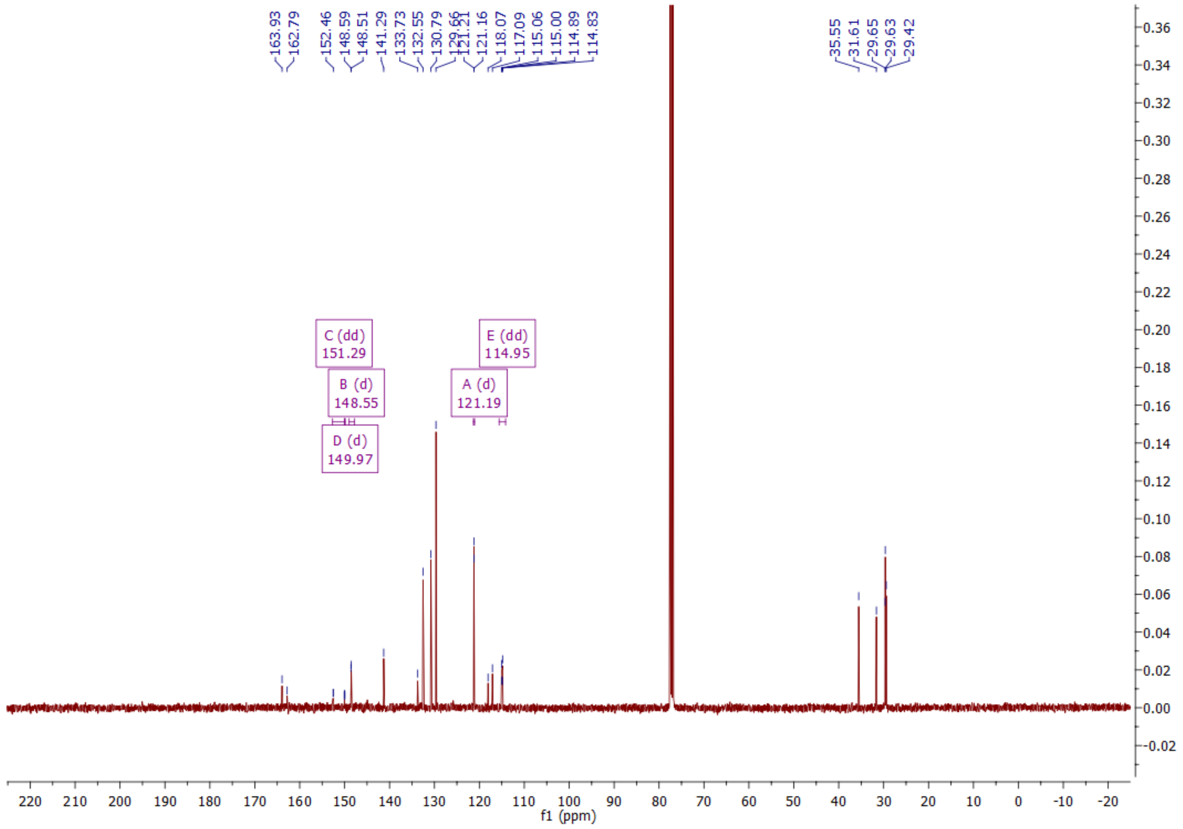

Figure SI-9: $^{13}C\{^{1}H\}$ (100.5 MHz, $CDCl_3$) NMR spectra of compound **9**

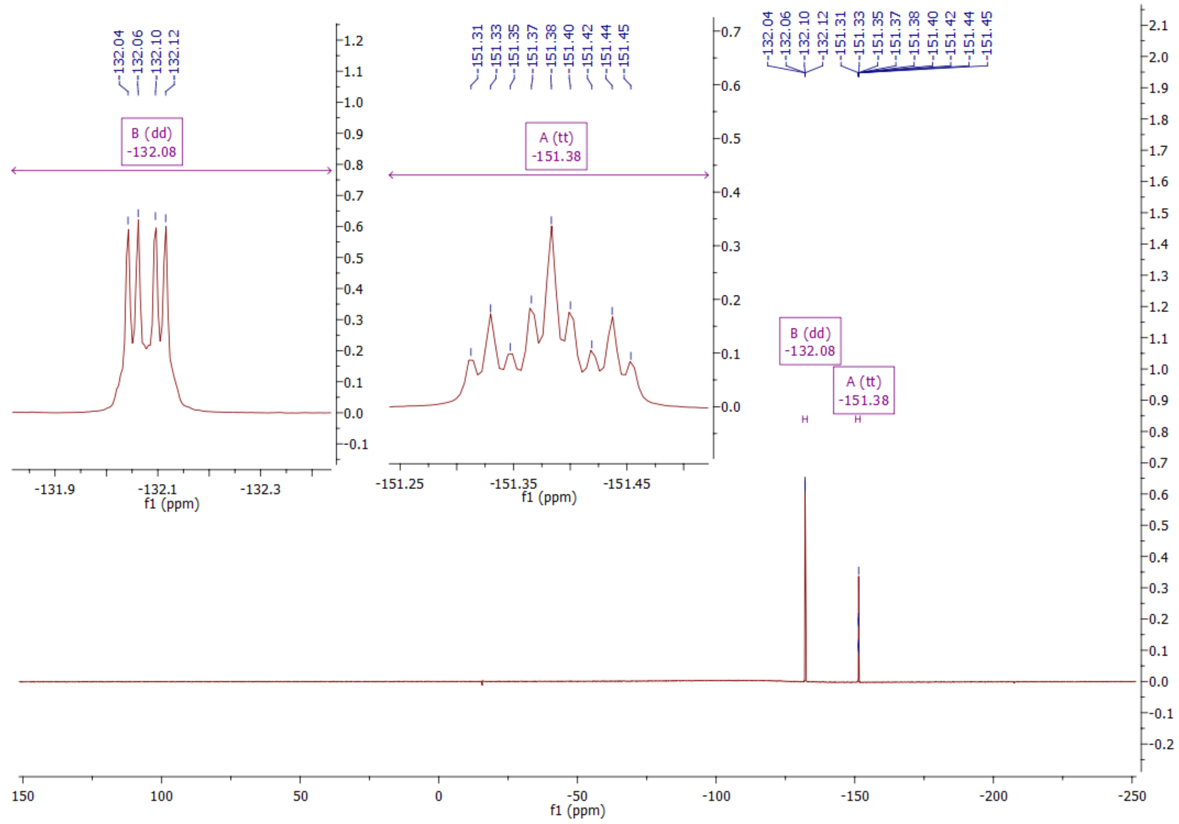

Figure SI-10: ^{19}F (376.4 MHz, $CDCl_3$) NMR spectra of compound **9**

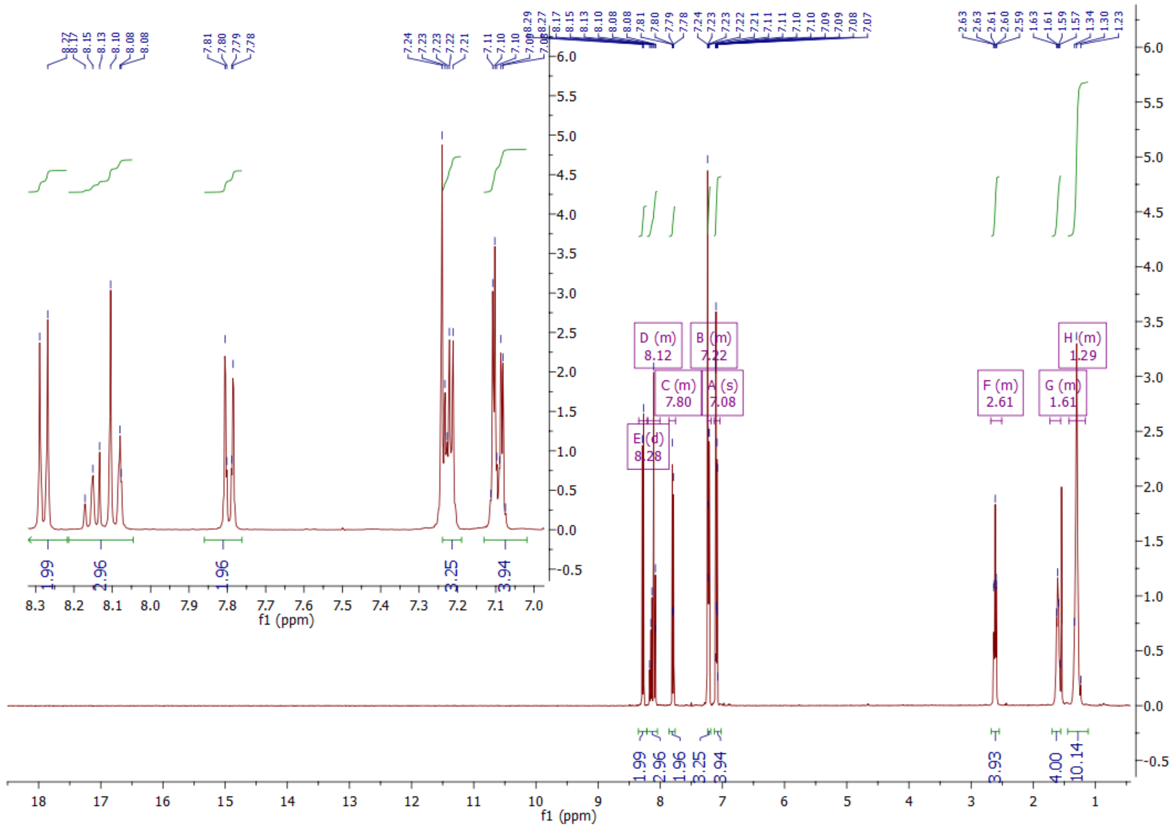

Figure SI-11: ^{1}H (400 MHz, $CDCl_3$) NMR spectra of compound **10**

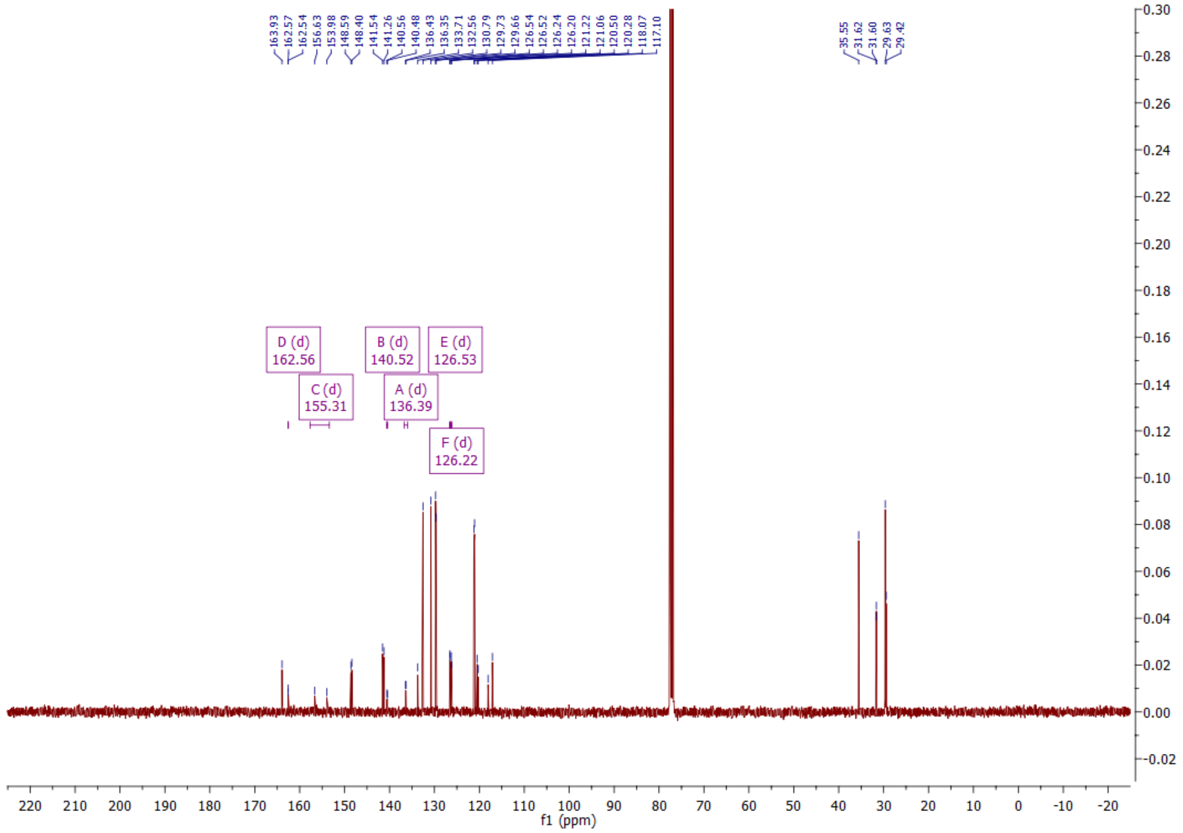

Figure SI-12: $^{13}C\{^{1}H\}$ (100.5 MHz, $CDCl_3$) NMR spectra of compound **10**

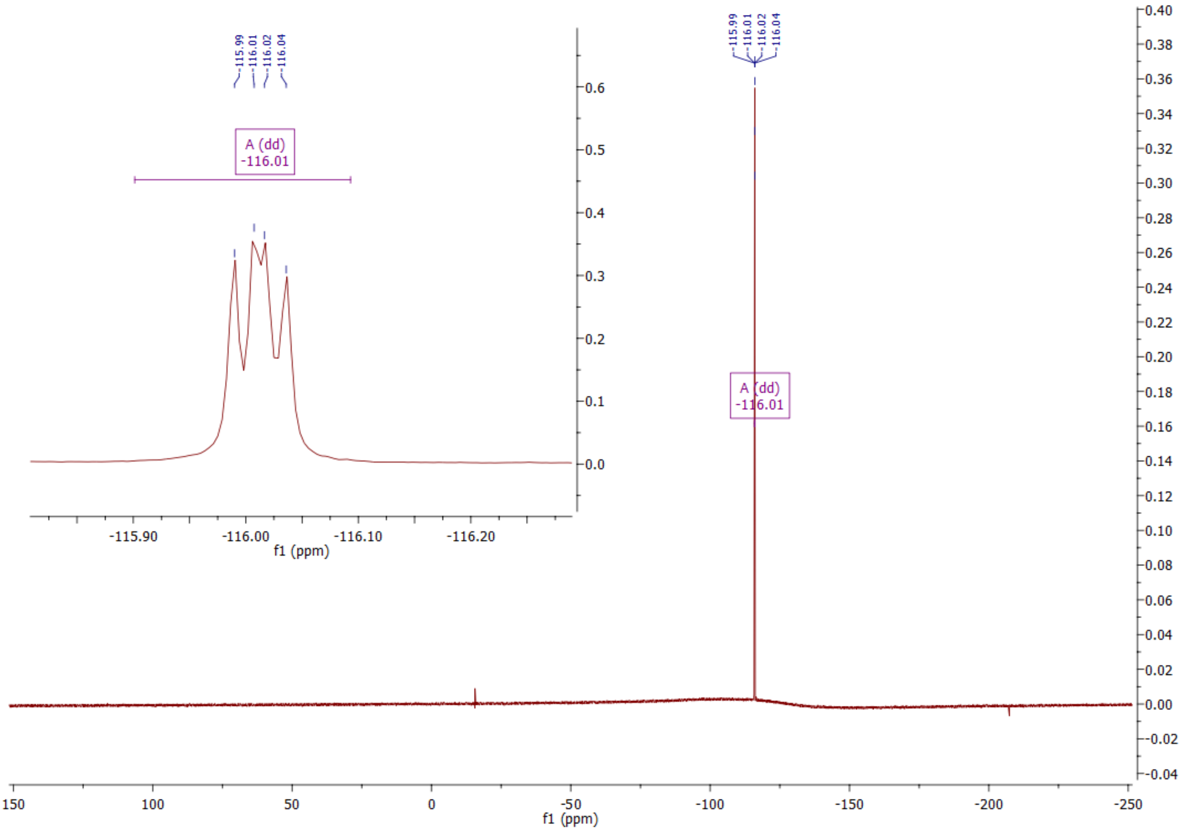

Figure SI-13: ^{19}F (376.4 MHz, $CDCl_3$) NMR spectra of compound **10**

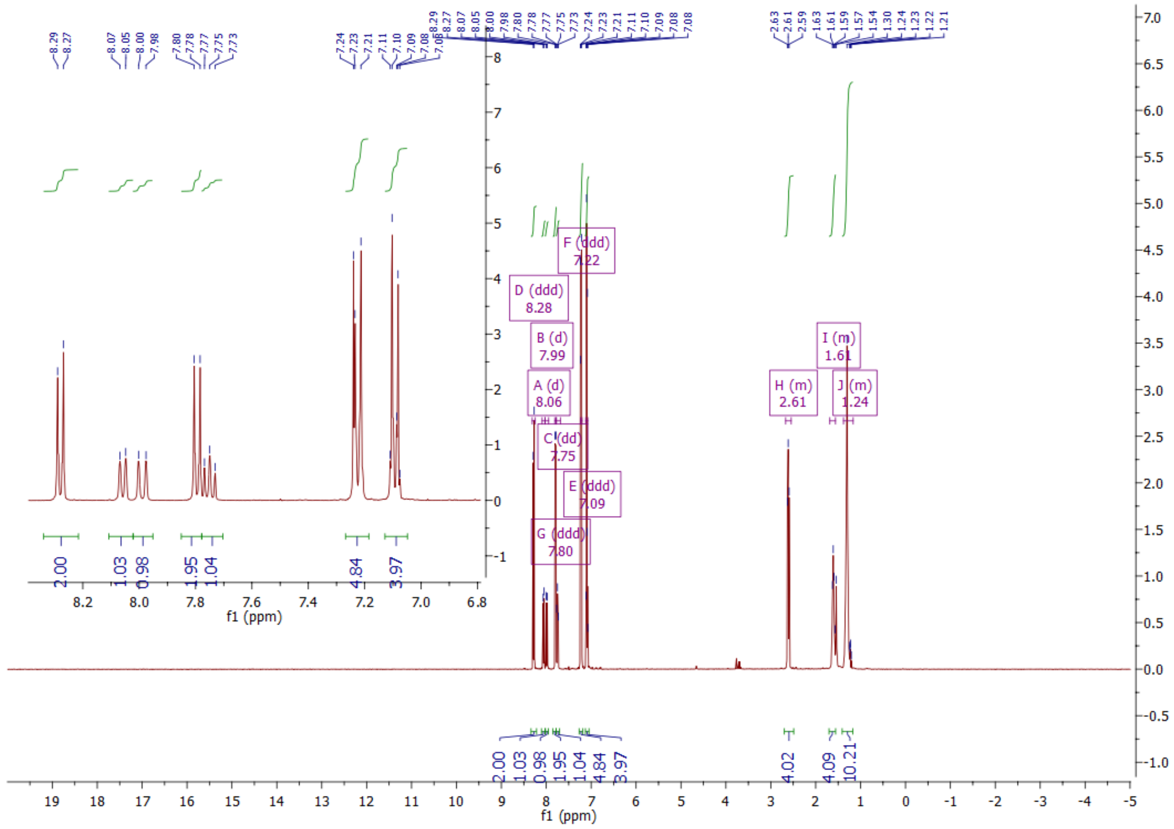

Figure SI-14: ^{1}H (400 MHz, $CDCl_3$) NMR spectra of compound **11**

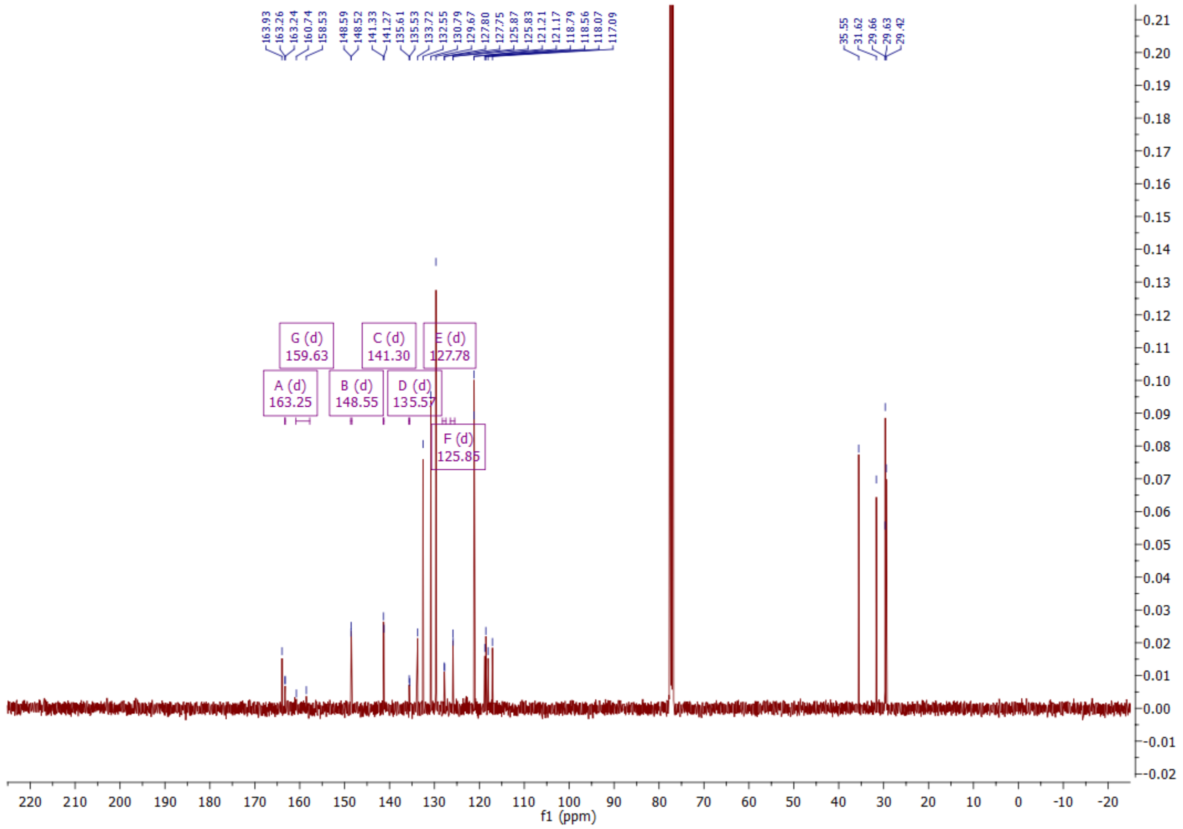

Figure SI-15: $^{13}C\{^1H\}$ (100.5 MHz, $CDCl_3$) NMR spectra of compound **11**

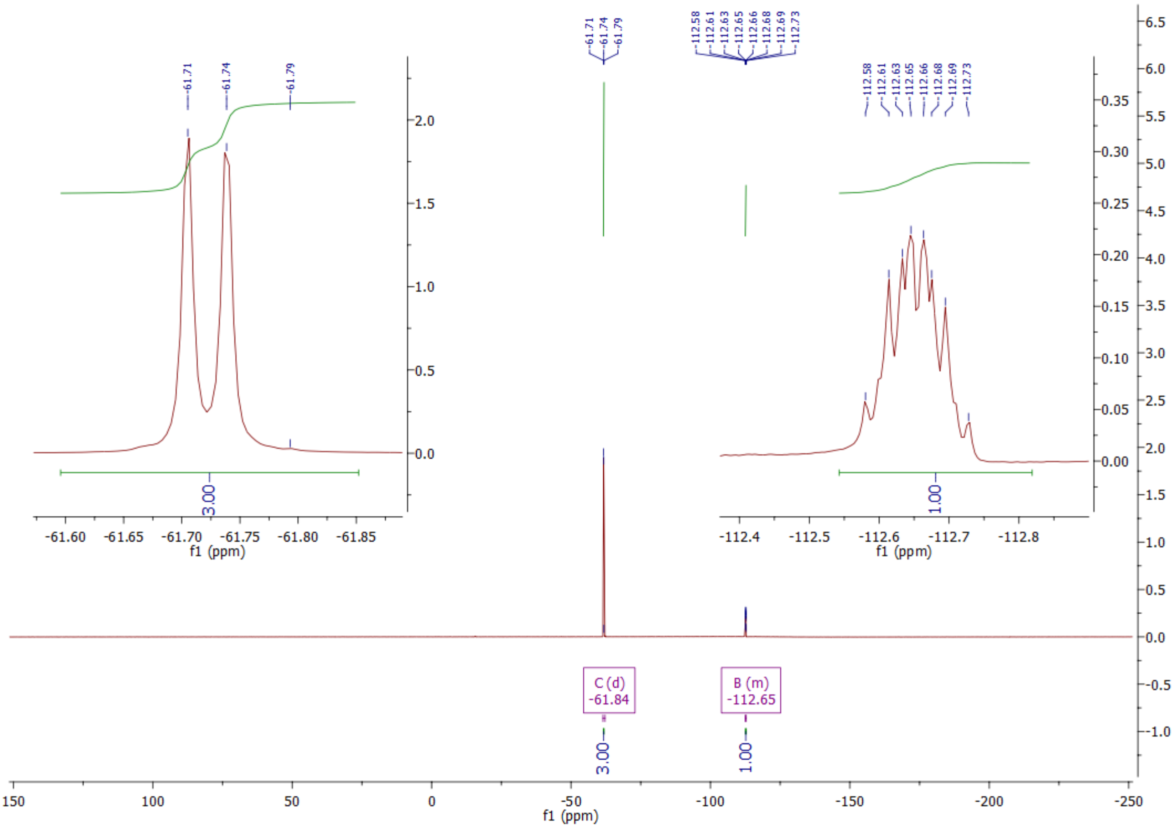

Figure SI-16: ^{19}F (376.4 MHz, $CDCl_3$) NMR spectra of compound **11**

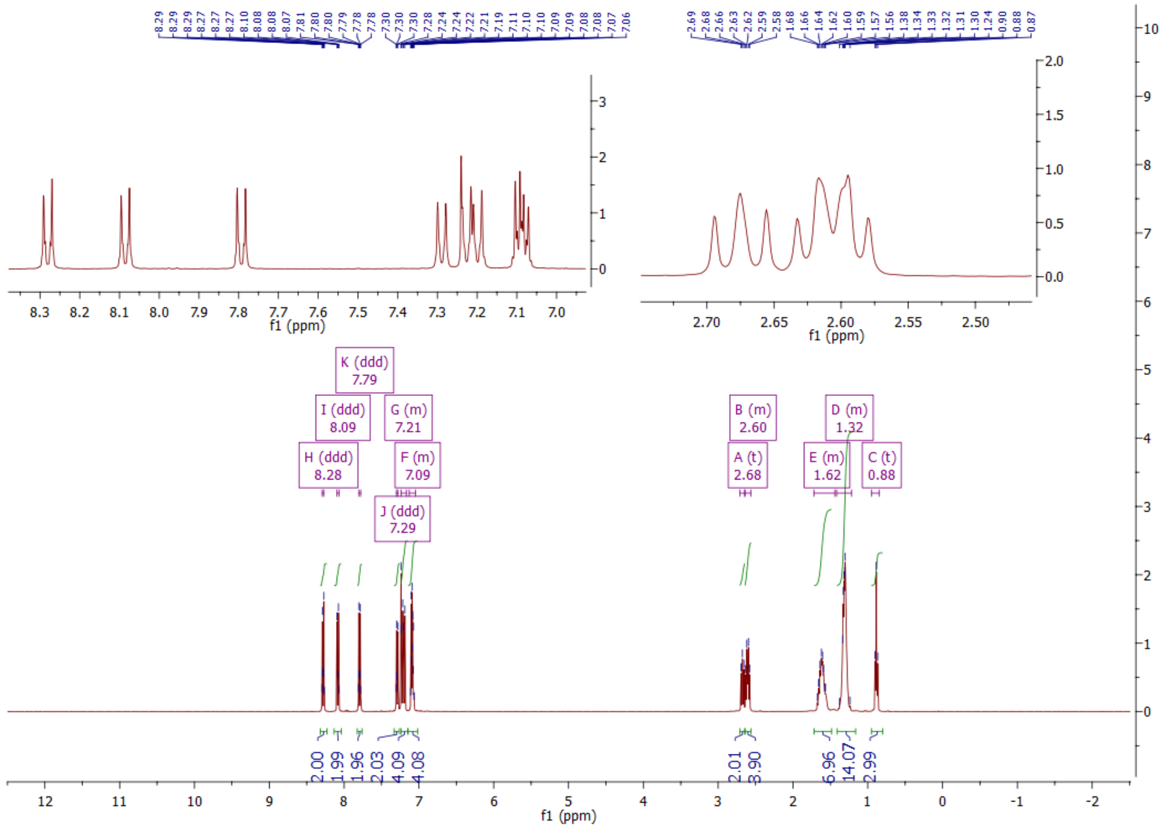

Figure SI-17: ^{1}H (400 MHz, $CDCl_3$) NMR spectra of compound **12**

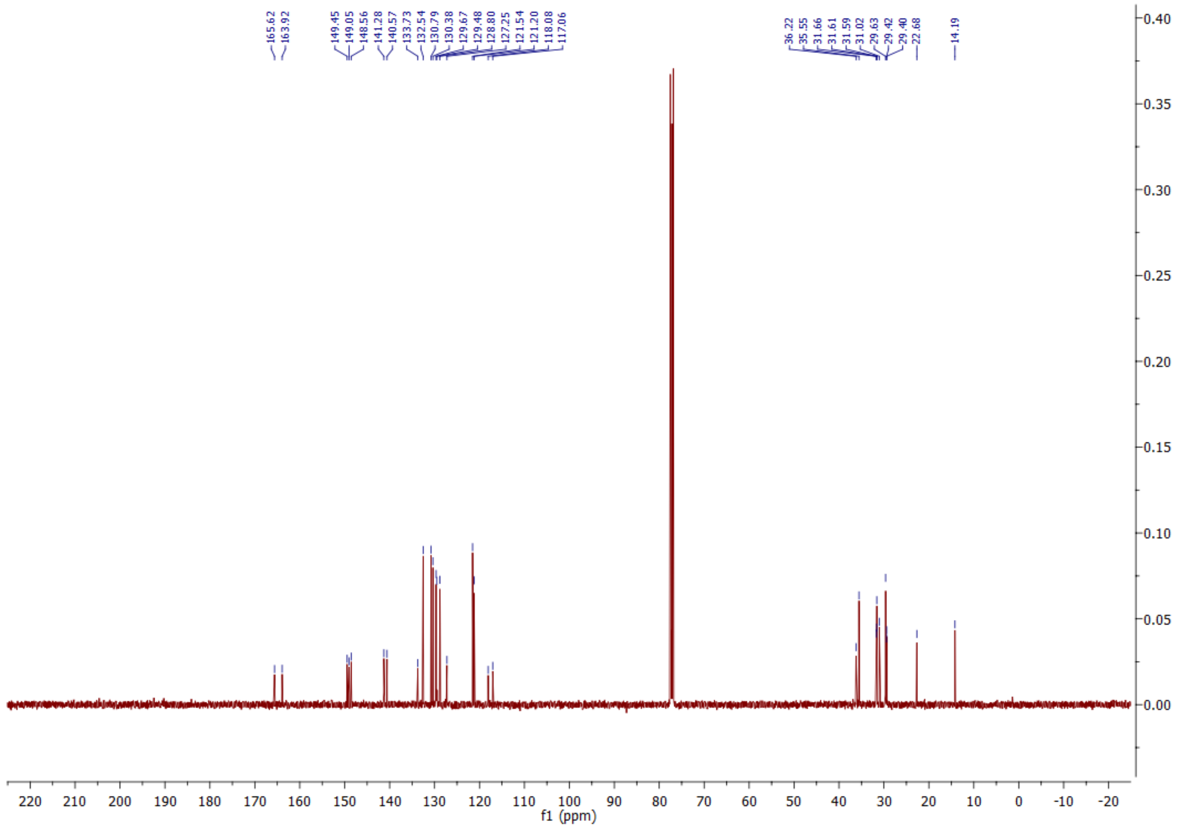

Figure SI-18: $^{13}C\{^{1}H\}$ (100.5 MHz, $CDCl_3$) NMR spectra of compound **12**

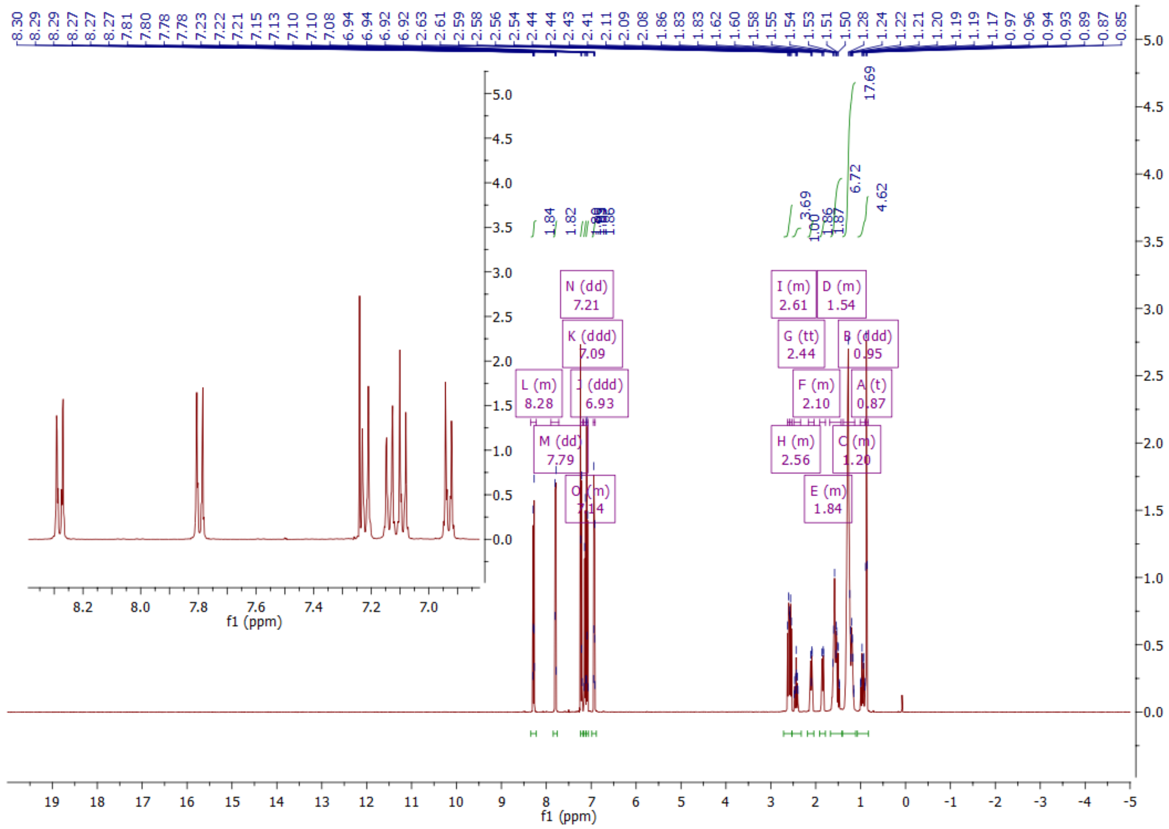

Figure SI-19: ^{1}H (400 MHz, $CDCl_3$) NMR spectra of compound **13**

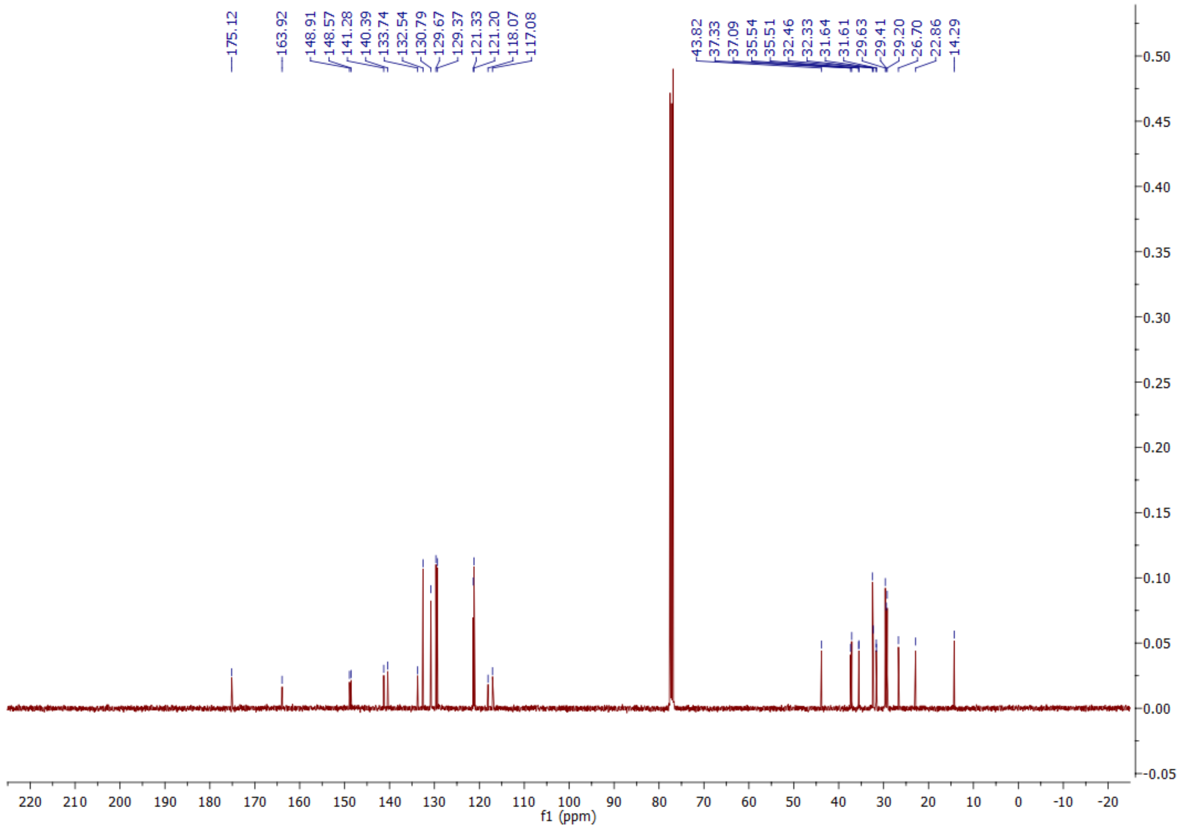

Figure SI-20: $^{13}C\{^{1}H\}$ (100.5 MHz, $CDCl_3$) NMR spectra of compound **13**

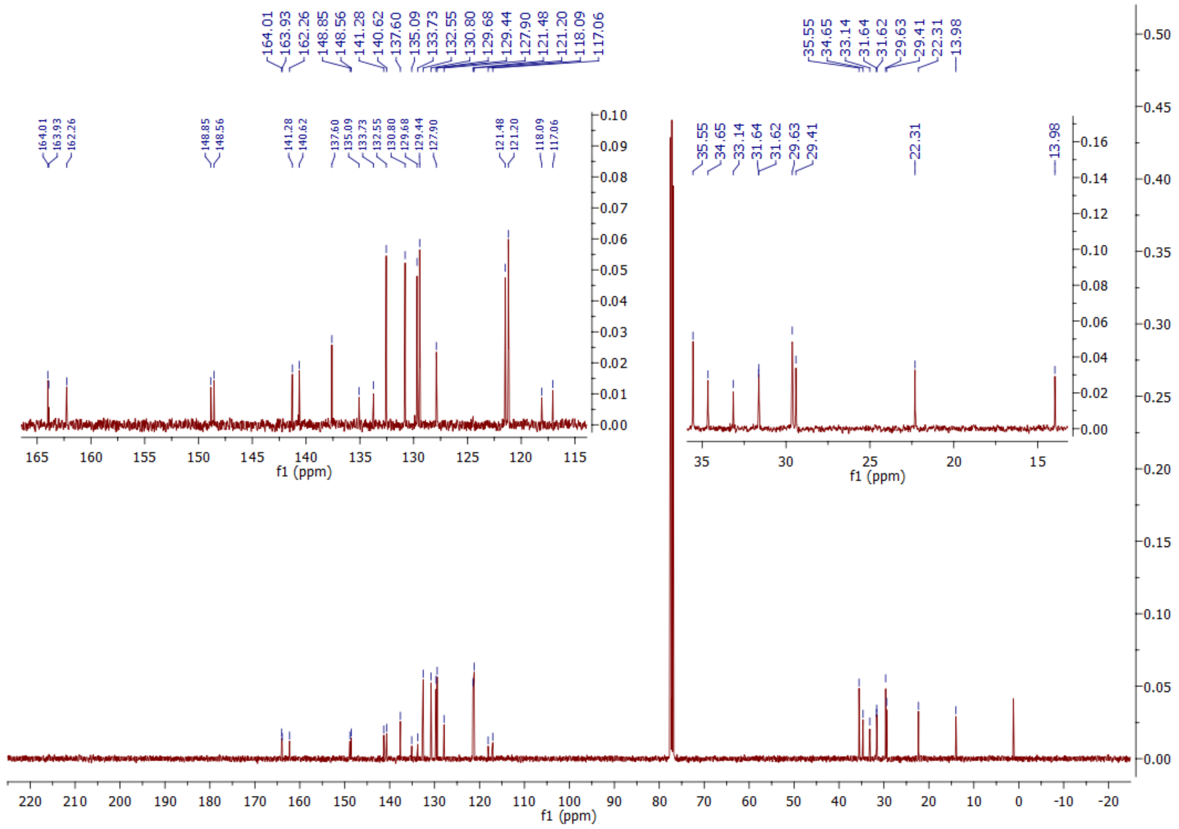

Figure SI-21: $^{13}C\{^{1}H\}$ (100.5 MHz, $CDCl_3$) NMR spectra of compound **14**

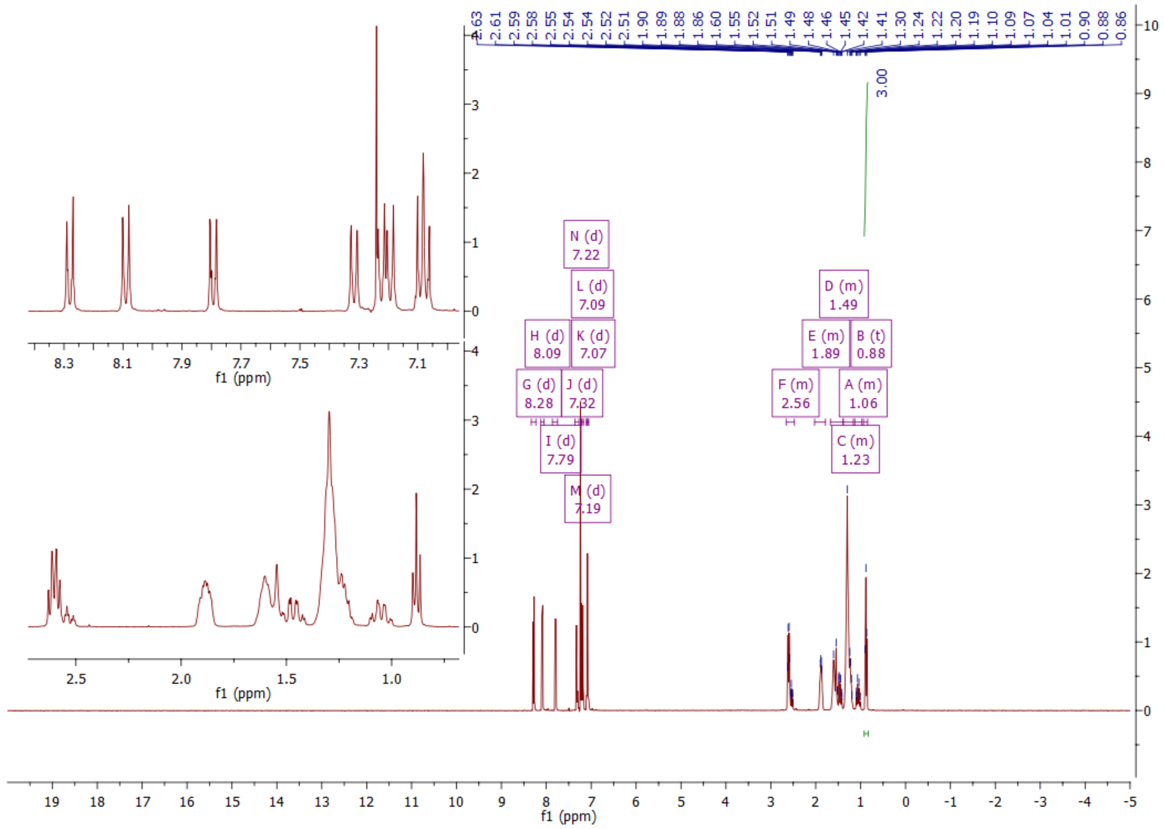

Figure SI-22: ^{1}H (400 MHz, $CDCl_3$) NMR spectra of compound **15**

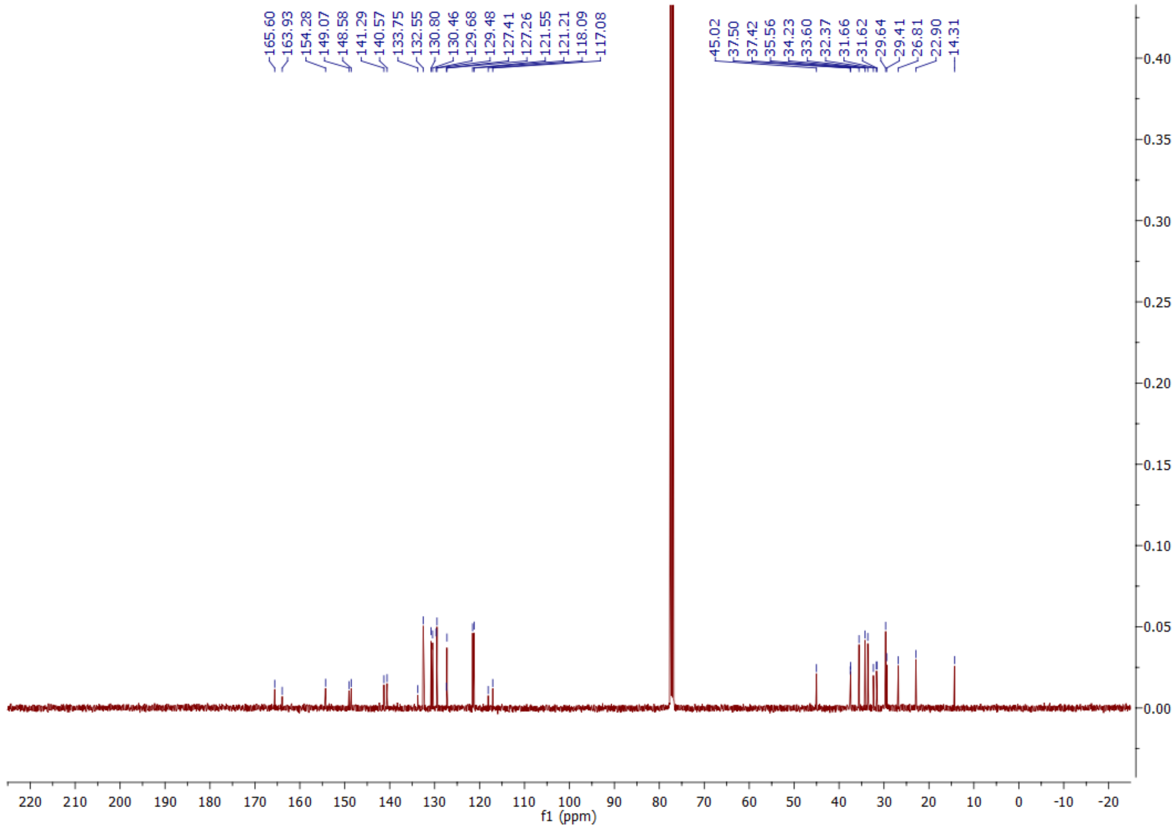

Figure SI-23: $^{13}C\{^1H\}$ (100.5 MHz, $CDCl_3$) NMR spectra of compound **15**

1.6. Tabulated DSC Data for Compounds 1 – 10

Cpd:	**1**	**2**	**3**	**4**	**5**	**6**	**7**	**8**	**9**	**10**	mean	stdev
**1**	1.72	1.72	1.72	1.72	1.73	1.73	1.74	1.74	1.73	1.73	1.73	0.008
**2**	0.70	0.70	0.69	0.69	0.69	0.70	0.69	0.70	0.70	0.70	0.70	0.005
**3**	0.29	0.29	0.29	0.30	0.29	0.29	0.28	0.29	0.29	0.29	0.29	0.003
**4**	1.02	1.01	1.01	1.01	1.01	1.02	1.02	1.02	1.02	1.01	1.02	0.005
**5**	0.32	0.32	0.32	0.31	0.31	0.31	0.32	0.32	0.31	0.31	0.32	0.003
**6**	1.10	1.06	0.96	0.81	1.04	0.93	0.90	1.07	1.00	0.83	0.97	0.102
**7**	1.31	1.32	1.31	1.32	1.31	1.31	1.32	1.31	1.31	1.32	1.31	0.004
**8**	0.25	0.24	0.25	0.24	0.24	0.24	0.24	0.24	0.24	0.24	0.25	0.002
**9**	0.65	0.65	0.65	0.65	0.65	0.65	0.65	0.65	0.65	0.65	0.65	0.003
**10**	0.74	0.74	0.74	0.74	0.74	0.74	0.74	0.74	0.74	0.74	0.74	0.002

Table SI-5: Tabulated DSC data for the nematic to isotropic transition of compounds **1 – 10**. Columns correspond to enthalpy (kJ mol^{-1}) measured for a given scan number (total of 10). Mean and standard deviations are given.

Cpd:	1	2	3	4	5	6	7	8	9	10	mean	stdev
**1**	0.41	0.40	0.40	0.40	0.41	0.40	0.41	0.41	0.41	0.40	0.40	0.005
**2**	0.25	0.24	0.24	0.25	0.25	0.25	0.24	0.25	0.24	0.25	0.24	0.003
**3**	0.11	0.12	0.00	0.11	0.17	0.10	0.06	0.11	0.07	0.08	0.09	0.042
**4**	0.03	0.03	0.30	0.27	0.01	0.03	0.03	0.05	0.02	0.03	0.07	0.109
**5**	0.48	0.47	0.47	0.47	0.41	0.48	0.48	0.49	0.48	0.48	0.47	0.022
**6**	0.44	0.45	0.57	0.42	0.64	0.53	0.66	0.60	0.50	0.00	0.48	0.189
**7**	0.15	0.14	0.14	0.15	0.14	0.14	0.15	0.14	0.15	0.14	0.14	0.006
**8**	0.30	0.30	0.24	0.19	0.23	0.30	0.31	0.31	0.31	0.30	0.25	0.044
**9**	0.73	0.72	0.74	0.72	0.72	0.71	0.73	0.72	0.72	0.72	0.72	0.007
**10**	0.23	0.24	0.23	0.24	0.24	0.24	0.24	0.24	0.24	0.24	0.24	0.003

Table SI-6: Tabulated DSC data for the nematic to N_{TB} transition of compounds **1 – 10**. Columns correspond to enthalpy (kJ mol^{-1}) measured for a given scan number (total of 10). Mean and standard deviations are given.

References

1. R. J. Mandle and J. W. Goodby, "A Liquid Crystalline Oligomer Exhibiting Nematic and Twist-Bend Nematic Mesophases", *ChemPhysChem,* **2016**, *7*, 967-970, DOI: 10.1002/cphc.201600038
2. R. J. Mandle and J. W. Goodby, "Progression from nano to macro science in soft matter systems: dimers to trimers and oligomers in twist-bend liquid crystals", *RSC Adv.,* **2016**, *6*, 34885-34893, DOI: 10.1039/C6RA03594A
3. R. J. Mandle, E. J. Davis, C.C.A.Voll, C. T. Archbold, J. W. Goodby and S. J. Cowling, "The Relationship between Molecular Structure and the Incidence of the N_{TB} Phase", *Liq. Cryst.,* **2015**, *42*, 688-703, DOI: 10.1080/02678292.2014.974698
4. Gaussian 09, Revision E.01, Frisch, M. J.; Trucks, G. W.; Schlegel, H. B.; Scuseria, G. E.; Robb, M. A.; Cheeseman, J. R.; Scalmani, G.; Barone, V.; Mennucci, B.; Petersson, G. A.; Nakatsuji, H.; Caricato, M.; Li, X.; Hratchian, H. P.; Izmaylov, A. F.; Bloino, J.; Zheng, G.; Sonnenberg, J. L.; Hada, M.; Ehara, M.; Toyota, K.; Fukuda, R.; Hasegawa, J.; Ishida, M.; Nakajima, T.; Honda, Y.; Kitao, O.; Nakai, H.; Vreven, T.; Montgomery, J. A., Jr.; Peralta, J. E.; Ogliaro, F.; Bearpark, M.; Heyd, J. J.; Brothers, E.; Kudin, K. N.; Staroverov, V. N.; Kobayashi, R.; Normand, J.; Raghavachari, K.; Rendell, A.; Burant, J. C.; Iyengar, S. S.; Tomasi, J.; Cossi, M.; Rega, N.; Millam, J. M.; Klene, M.; Knox, J. E.; Cross, J. B.; Bakken, V.; Adamo, C.; Jaramillo, J.; Gomperts, R.; Stratmann, R. E.; Yazyev, O.; Austin, A. J.; Cammi, R.; Pomelli, C.; Ochterski, J. W.; Martin, R. L.; Morokuma, K.; Zakrzewski, V. G.; Voth, G. A.; Salvador, P.; Dannenberg, J. J.; Dapprich, S.; Daniels, A. D.; Farkas, Ö.; Foresman, J. B.; Ortiz, J. V.; Cioslowski, J.; Fox, D. J. Gaussian, Inc., Wallingford CT, 2009.
5. Z. Zhang, V. P. Panov, M. Nagaraj, R. J. Mandle, J. W. Goodby, G. R. Luckhurst, J. C. Jones and h. F. Gleeson, "Raman scattering studies of order parameters in liquid crystalline dimers exhibiting the nematic and twist-bend nematic phases", *J. Mater. Chem. C,* **2015**, *3*, 10007 – 10016.
6. C. T. Archbold, J. L. Andrews, R. J. Mandle, S. J. Cowling and J.W. Goodby, "Effect of the Linking Unit on the Twist-Bend Nematic Phase in Liquid Crystal Dimers: A Comparative Study of Two Homologous Series of Methylene and Ether Linked Dimers.", *Liq. Cryst.,* **2016**,.
